# Mapping the evolution and research landscape of ferroptosis-targeted nanomedicine: insights from a scientometric analysis

**DOI:** 10.3389/fphar.2024.1477938

**Published:** 2024-09-25

**Authors:** Siyang Cao, Yihao Wei, Yaohang Yue, Deli Wang, Jun Yang, Ao Xiong, Hui Zeng

**Affiliations:** ^1^ National and Local Joint Engineering Research Centre of Orthopaedic Biomaterials, Peking University Shenzhen Hospital, Shenzhen, Guangdong, China; ^2^ Shenzhen Key Laboratory of Orthopaedic Diseases and Biomaterials Research, Peking University Shenzhen Hospital, Shenzhen, Guangdong, China; ^3^ Department of Bone and Joint Surgery, Peking University Shenzhen Hospital, Shenzhen, Guangdong, China; ^4^ Department of Rehabilitation Science, The Hong Kong Polytechnic University, Hong Kong Special Administrative Region, Hong Kong, China; ^5^ Faculty of Pharmaceutical Sciences, Shenzhen Institute of Advanced Technology, Chinese Academy of Sciences (CAS), Shenzhen, Guangdong, China; ^6^ Department of Radiology, Peking University Shenzhen Hospital, Shenzhen, Guangdong, China; ^7^ Department of Orthopedics, Shenzhen Second People’s Hospital, The First Affiliated Hospital of Shenzhen University, Shenzhen, Guangdong, China

**Keywords:** global research trend, ferroptosis, nano drug delivery systems, scientometrics, visualized analysis

## Abstract

**Objective:**

Notable progress has been made in “ferroptosis-based nano drug delivery systems (NDDSs)” over the past 11 years. Despite the ongoing absence of a comprehensive scientometric overview and up-to-date scientific mapping research, especially regarding the evolution, critical research pathways, current research landscape, central investigative themes, and future directions.

**Methods:**

Data ranging from 1 January 2012, to 30 November 2023, were obtained from the Web of Science database. A variety of advanced analytical tools were employed for detailed scientometric and visual analyses.

**Results:**

The results show that China significantly led the field, contributing 82.09% of the total publications, thereby largely shaping the research domain. Chen Yu emerged as the most productive author in this field. Notably, the journal *ACS Nano* had the greatest number of relevant publications. The study identified liver neoplasms, pancreatic neoplasms, gliomas, neoplasm metastases, and melanomas as the top five crucial disorders in this research area.

**Conclusion:**

This research provides a comprehensive scientometric assessment, enhancing our understanding of NDDSs focused on ferroptosis. Consequently, it enables rapid access to essential information and facilitates the extraction of novel ideas in the field of ferroptotic nanomedicine for both experienced and emerging researchers.

## 1 Introduction

**FIGURE 1:**
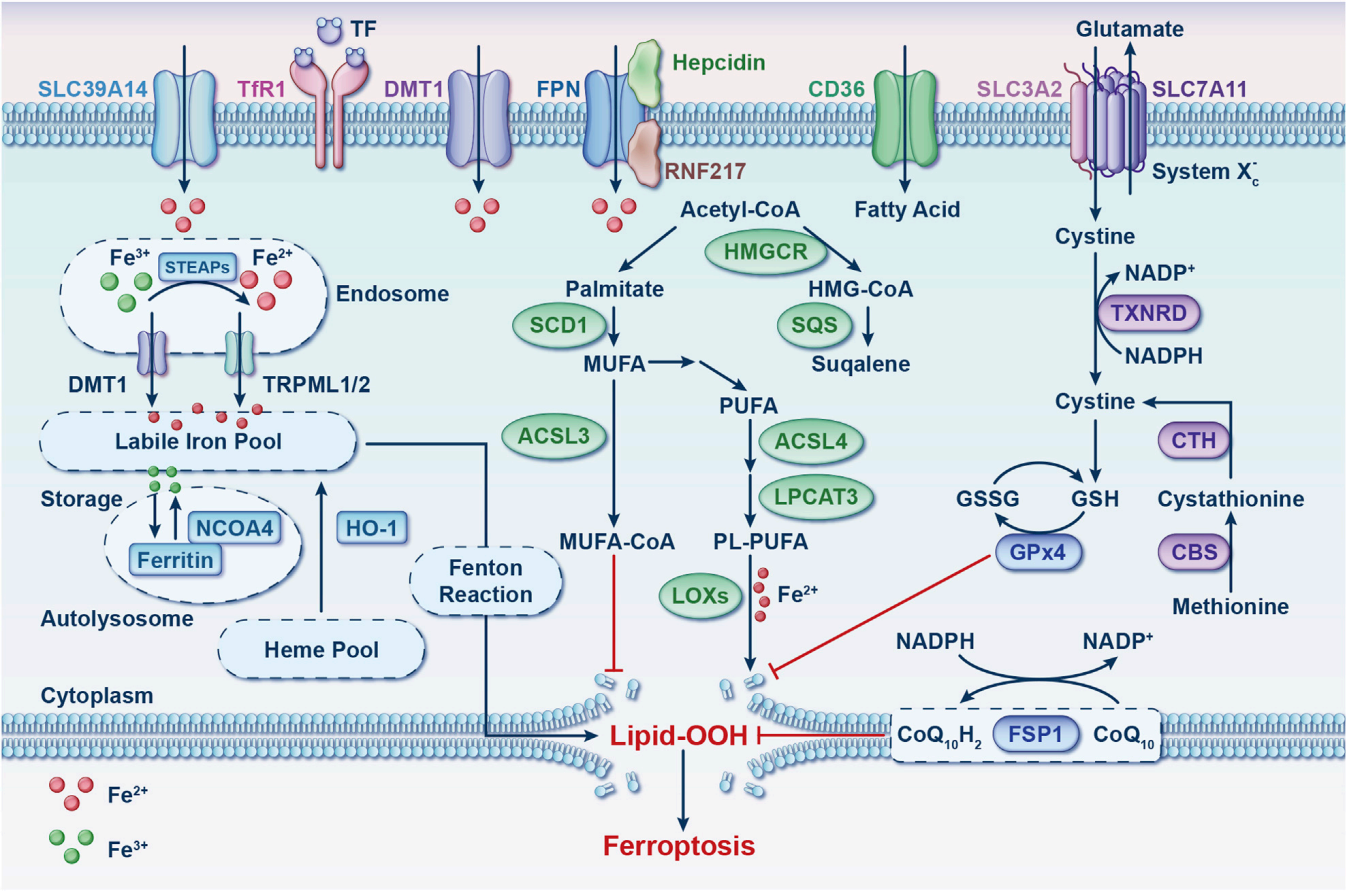
Ferroptosis: molecular mechanisms. This process involves three pathways: iron metabolism, redox processes, and lipid metabolism. Imbalances in oxidative-reductive systems, iron metabolism, or polyunsaturated fatty acid peroxidation can trigger ferroptosis. Abbreviations: ACSL3, acyl-CoA synthetase long-chain family member 3; ACSL4, acyl-CoA synthetase long-chain family member 4; CBS, cystathionine beta-synthase; CD36, cluster differentiation 36; CoQ_10_, coenzyme Q_10_; CTH, cystathionine gamma-lyase; DMT1, divalent metal transporter 1; FPN, ferroportin; FSP1, ferroptosis suppressor protein 1; GPx4, glutathione peroxidase 4; GSH, glutathione; GSSG, glutathione disulfide; HMGCR, 3-hydroxy-3-methylglutaryl-CoA reductase; HMG-CoA, 3-hydroxy-3-methylglutaryl-CoA; HO-1, heme oxygenase 1; LOXs, lipoxygenases; LPCAT3, lysophosphatidylcholine acyltransferase 3; MUFA, monounsaturated fatty acid; NADP^+^, nicotinamide-adenine dinucleotide phosphate; NADPH, reduced form of nicotinamide-adenine dinucleotide phosphate; NCOA4, nuclear receptor coactivator 4; PL-PUFA, phospholipid-containing polyunsaturated fatty acid; PUFA, polyunsaturated fatty acid; RNF217, E3 ubiquitin protein ligase RNF217; SCD1, stearoyl-coenzyme A desaturase 1; SLC3A2, solute carrier family three member 2; SLC7A11, solute carrier family seven member 11; SLC39A14, solute carrier family 39 member 4; SQS, squalene synthase; STEAP, 6-transmembrane epithelial antigen of the prostate metalloreductase family; TF, transferrin; TfR1, transferrin receptor 1; TRPML, lysosomal cation channel mucolipin; TXNRD, thioredoxin reductase.

Ferroptosis, initially identified through RAS-selective-lethal (RSL) compound screening, is an iron-dependent programmed cell death (PCD) process (Dixon et al., 2012). Its hallmark is the substantial accumulation of lipid peroxides, which distinguishes it genetically, morphologically, and biochemically from other forms of PCD (Dixon et al., 2012). Over the past 11 years, extensive research has emphasized ferroptosis’s vital role in biological processes such as tumor inhibition and immune response modulation, underscoring its significance in maintaining health through iron/lipid metabolism and glutathione (GSH)-dependent redox homeostasis (Figure 1) (Sun et al., 2023; Stockwell, 2022).

**FIGURE 2:**
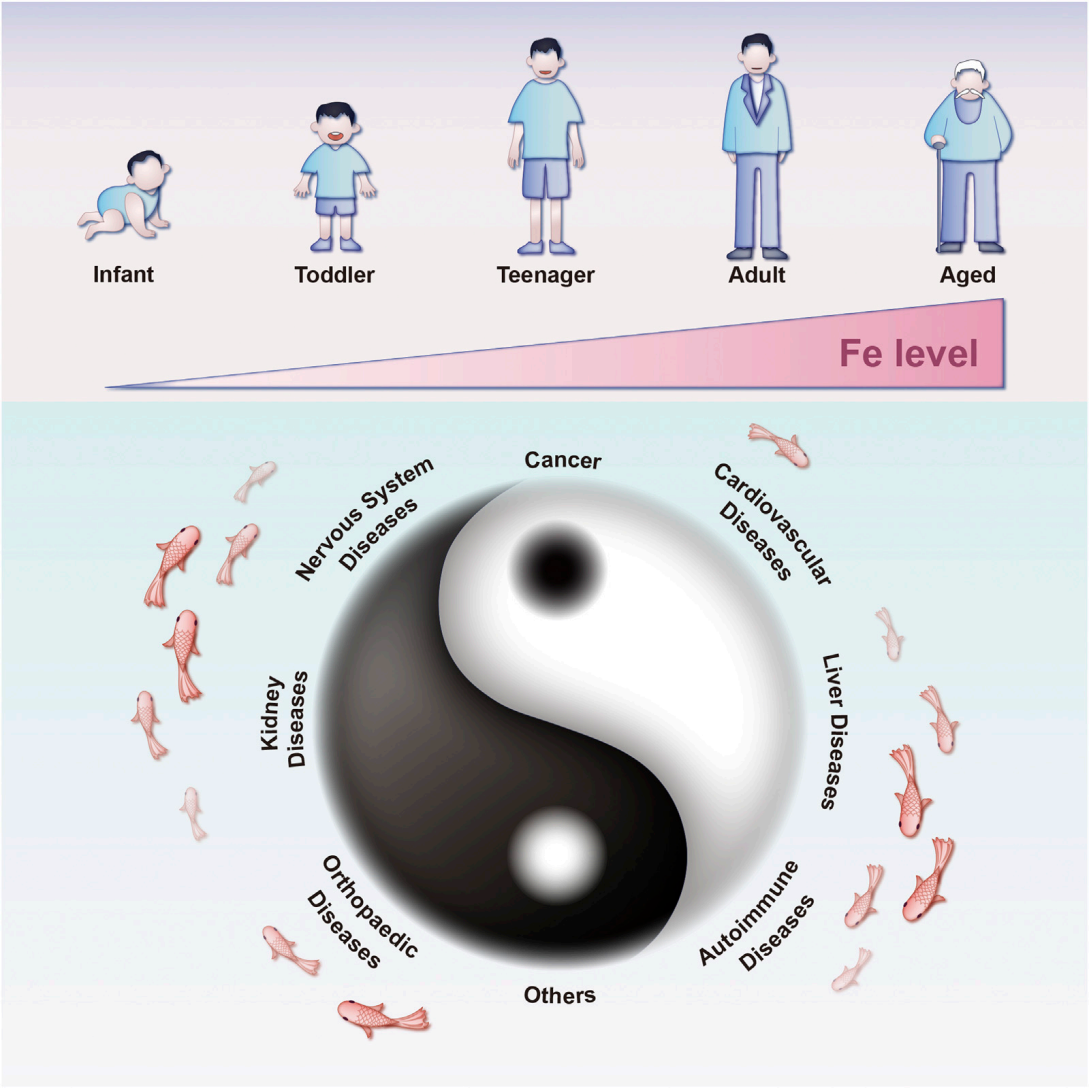
Diseases associated with ferroptosis throughout the human lifespan. Iron accumulation due to aging can initiate ferroptosis, increasing the risks of hypoxic-ischemic brain injury, organ damage, and neurodegenerative diseases. Reduced ferroptosis can contribute to the development of diverse cancers across different life stages.

An abundance of iron in cells has been noted to increase their susceptibility to ferroptosis (Fang et al., 2019; Yang et al., 2020; Yu et al., 2020; Fang et al., 2020). Recent studies have unveiled that aging individuals experience cellular iron accumulation, potentially leading to diverse tissue degenerative conditions and diseases (Figure 2A) (Ru et al., 2023). Thus, preserving iron homeostasis is essential for protecting cells from ferroptosis. Lipid metabolism, especially that of polyunsaturated fatty acids (PL-PUFAs), occupies a significant place in regulating ferroptosis (Stockwell, 2022; Liang et al., 2022). The buildup of phospholipid (PL) peroxides in cell membranes, primarily due to free iron-initiated Fenton reactions and the oxidation of PLs containing PL-PUFAs, directly initiates ferroptosis (Dixon et al., 2012; Stockwell et al., 2017). In the realm of oxidative and reductive reactions, key pathways that provide defense against ferroptosis have been identified (Figure 1). The cystine/glutamate antiporter system (system Xc^−^)-GSH-glutathione peroxidase 4 (GPx4) axis is chiefly acknowledged for its ability to scavenge PL peroxides in a GPx4-dependent manner, facilitated by GSH synthesis through the system Xc^−^ (Dixon et al., 2012; Yang et al., 2014). Interfering with components of system Xc^−^ activates ferroptosis by obstructing cystine uptake, which in turn restricts GSH synthesis (Dixon et al., 2012). GPx4 plays a pivotal role as a regulator within this pathway (Yang et al., 2014).

Ferroptosis has been increasingly considered to mediate the pathogenesis and development of multiple diseases (Figure 2B), including cancer, nervous system diseases, cardiovascular diseases, hepatopathy, nephropathy, musculoskeletal diseases, autoimmune diseases, and others (Wang X. et al., 2023). Given the therapeutic potential inherent in the regulation of ferroptosis, it would be of far-reaching clinical significance to hone ferroptosis-mediating therapeutics for treating various diseases. On one hand, in the field of oncology, ferroptosis-inducing agents could be used to induce PCD and eliminate malignant cells (Friedmann et al., 2019; Ajoolabady et al., 2023; Sui et al., 2022; Wu S. et al., 2021; Mou et al., 2019). Conversely, ferroptosis inhibitors has the potential to alleviate cytotoxic damage in healthy cells induced by abnormal metabolism of iron, GSH, and lipids (Ru et al., 2023; Cao et al., 2023a; Cao et al., 2023b; Fang et al., 2023; Wu X. et al., 2021; Wang ZL. et al., 2022; Lane et al., 2021). Both unity and opposition are embodied by this characteristic, which is a perfect fit for ancient Chinese philosophical concept of Yin and Yang (Figure 2B).

**FIGURE 3:**
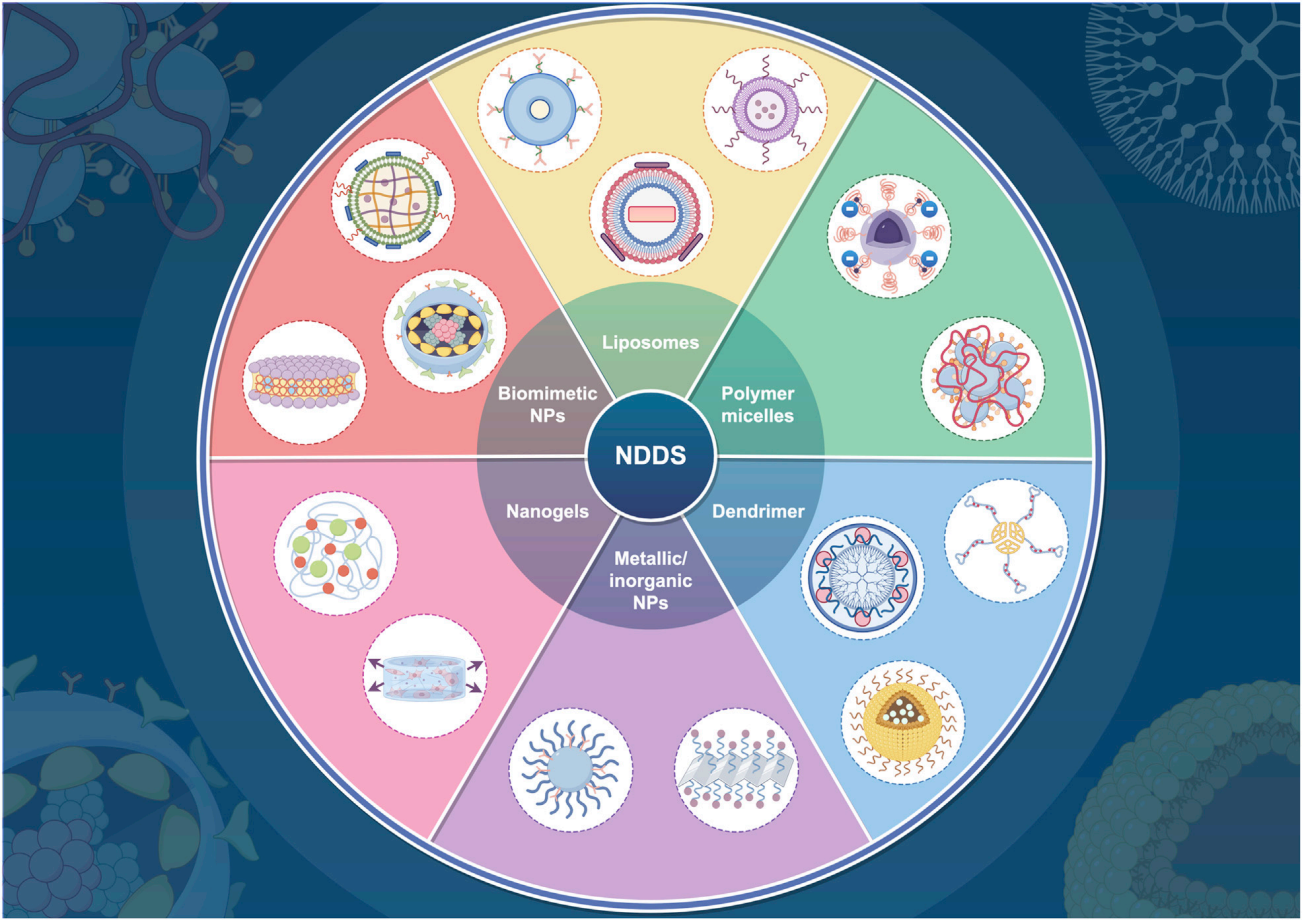
Various nanocarrier types utilized in drug delivery systems. This figure was created using Figdraw (https://www.figdraw.com/static/index.html#/). Abbreviations: NDDS, nano drug delivery system; NPs, nanoparticles.

Recent advancements in the sphere of ferroptosis inducers and inhibitors have shown a significant increase, with numerous methods being explored for disease treatment through the induction or inhibition of ferroptosis. This includes the use of various small molecules, notably erastin (Chen et al., 2021a), RSL3 (Yang et al., 2014), auranofin (Yang et al., 2020), ferrostatin-1 (Dixon et al., 2012), rosiglitazone (Wang et al., 2022b), and liproxstatin-1 (Zilka et al., 2017). Despite these developments, the poor pharmacokinetics and relatively low bioavailability of current ferroptosis-involved drugs pose substantial challenges to their broader clinical application (Sun et al., 2023; Liu Q. et al., 2023; Li et al., 2021). Ferroptosis, not exclusive to tumor cells, also affects normal cells and tissues. Consequently, administering ferroptosis molecular inducers *in vivo* without tumor-targeting capabilities can lead to systemic adverse reactions. The challenges of large-scale *in vivo* application pose significant barriers to clinical translation (Cai et al., 2023). The evolution of nanotechnology has fortunately brought forth nano drug delivery systems (NDDSs) characterized by designability, biocompatibility, multifunctionality, and modifiability (Figure 3) (Zhang S. et al., 2022; Shan et al., 2020; Wong et al., 2020; Wang J. et al., 2021; Zhang C. et al., 2020; Zheng H. et al., 2021). These attributes offer significant benefits to nano-systems in pharmaceutical applications related to ferroptosis, including passive or active targeted delivery, stimuli-responsive properties (endogenous, exogenous, or multiplexed), organism-wide dispersion, upgraded drug solubility, and the potential for combination therapies (Jiang and Zhang, 2023; Cook and Decuzzi, 2021; Ovais et al., 2020; Chu et al., 2023; Mao et al., 2023; Moradi Kashkooli et al., 2020).

For the rapid development of ferroptosis and nanotechnology (Liu Q. et al., 2023; Saleem et al., 2018; Qiao et al., 2022; Yang H. et al., 2023), despite various reviews on “ferroptosis-based NDDSs” (Liu Q. et al., 2023; Cai et al., 2023; Zhang S. et al., 2022; Zheng H. et al., 2021; Qiao et al., 2022; Luo et al., 2021; Yao et al., 2023; Wang H. et al., 2023; Zhang et al., 2021), these assessments frequently lack substantiation through objective visualized data. Instead, they heavily depend on the subjective comprehension of the disciplinary framework by researchers. This variability and subjectivity hinder comprehensive analysis, identification of research focal points, and determination of cutting-edge directions. To address these limitations, the current investigation used scientometric analysis to visually depict publications, nations/regions, authors, organizations, keywords, references, and journals in “ferroptosis-based NDDSs” over the past 11 years. The objectives included examining research output distribution, recognizing primary contributors, pinpointing hotspots, evaluating the current status, and exploring frontiers. In the event that such a comprehensive and systematic knowledge base is established, researchers from a variety of disciplines will find it easier to navigate the domain’s breadth. In addition, it guides scholars entering the field into interesting research directions. To our knowledge, the present investigation represents the initial comprehensive scientometric analysis of this subject.

## 2 Methods and methods

### 2.1 Data source and retrieval strategy

The Web of Science Core Collection (WoSCC) (https://www.webofscience.com/wos/) tracks scientific frontiers’ evolution, allowing researchers to thoroughly analyze and comprehend trends in academic publications (Boudry et al., 2018; Zhang XL. et al., 2020; Pei et al., 2022; Ling et al., 2023). WoSCC serves as a crucial platform, offering bibliometric software for general statistics (Pei et al., 2022), and demonstrating superior accuracy in labeling document types compared to other databases (Yeung, 2019). This study encompassed an exhaustive online exploration within the WoSCC, concentrating on original research and reviews related to “ferroptosis-based NDDSs” for targeted therapy. The inquiry covered publications from 1 January 2012, to 30 November 2023, utilizing both Medical Subject Heading terms and free words for data retrieval. To enhance the sensitivity and precision of the search strategy, three researchers (SYC, YHW, and YHY) conducted multiple iterations for its refinement. In the Supplementary Material, the search methodology is extensively explained.

### 2.2 Inclusion and exclusion criteria

The inclusion criteria focused on investigations related to “ferroptosis-based NDDSs”, specifically original research articles and reviews published in English. Exclusion criteria encompassed dissertations, letters, commentaries, editorials, conference abstracts, and studies from journals with similar or different titles. Two authors (SYC and YHW) independently conducted this process and cross-verified the results, with any discrepancies resolved by a senior author (DLW).

### 2.3 Statistical analysis

Data were sourced from WoSCC and processed using various software tools: Microsoft Excel (Office 365, Microsoft) for data organization, VOSviewer 1.6.18 (Leiden University, Netherlands) and Pajek 64 5.16 (University of Ljubljana, Slovenia) for co-occurrence analysis, Citespace version 6.2.6R (Chaomei Chen, China) for visual mapping, Scimago Graphica version 1.0.35 (https://www.graphica.app/, United States) for graphical analysis, and the chorddiag R package (R Studio, version 4.2.0) for specialized graphics.

Using chorddiag, Scimago Graphica, and VOSviewer, national/regional collaboration maps and charts were created for publication analysis. VOSviewer and Pajek were used for co-occurrence analyses across institutions, authors, journal publications, keywords, and diseases. The utilization of Citespace involved the analysis and mapping of data pertaining to countries/regions, institutions, authors, journals, co-citations, and keywords. Additionally, charts depicting the top 10 emergence intensities for countries/regions, institutions, authors, and keywords, along with the top 20 citation emergence intensities for journals and literature, were generated.

Citexs Data Analysis Platform (https://www.citexs.com) was utilized to gather disease information, generating relevant visual graphs to deliver a comprehensive evaluation of the current state, key focal points, and emerging trends in this domain.

## 3 Results

### 3.1 Scientific output

**FIGURE 4:**
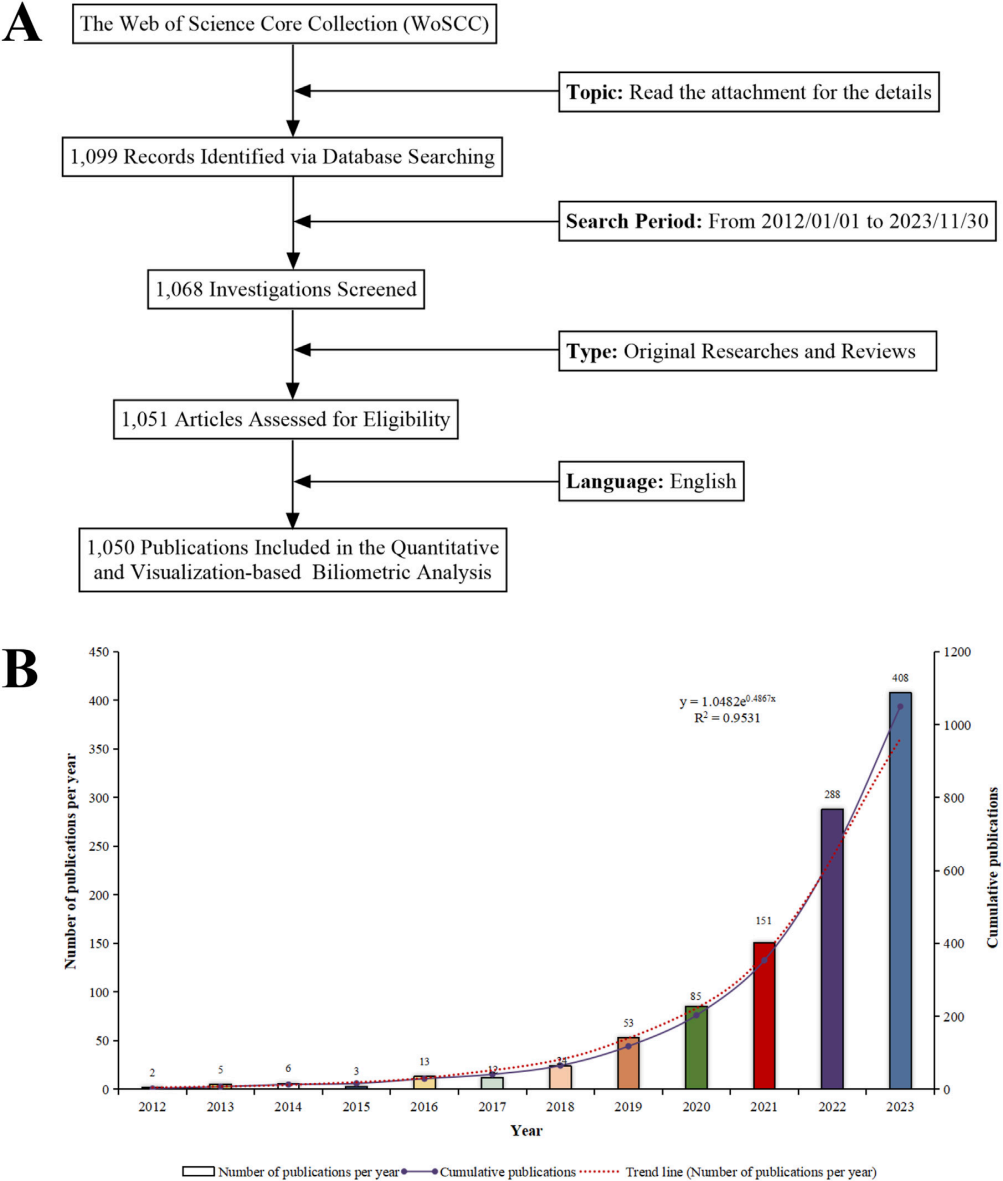
**(A)** Schematic of the literature search and selection process. **(B)** Trend analysis of research on “ferroptosis-nano drug delivery systems” (2012–2023).

In Figure 4A, data retrieval and collection are illustrated. The research progress is indicated by the number of scientific investigations produced within a specific period (Koo, 2021). Between 2012 and 2023, we assembled 1,050 pertinent scientific reports concerning “ferroptosis-based NDDSs”, comprising 881 original articles and 169 reviews, averaging 95.45 publications annually. During the past 11 years, academic output in this field has steadily increased. In 2021, the annual count of relevant publications surpassed 100, peaking at 408. With an annual growth rate of 85.08%, this surge represents more than 200-fold growth since 2012. Research activity in this field continues to rise and its significance for research is evident from this trend. An exponential equation (y = 1.0482e^0.4867x^, where x is the year and y the yearly publication count, *R* ^2^ = 0.9531) was applied to fit the annual pattern, resulting in a well-fitted curve (Figure 4B). The expansion of research environments contributes to the accumulation of extensive knowledge, enhancing both current and future research endeavors. There can therefore be expected to be significant advancements in research on “ferroptosis-based NDDSs” over the coming years.

### 3.2 Countries/regions

**FIGURE 5:**
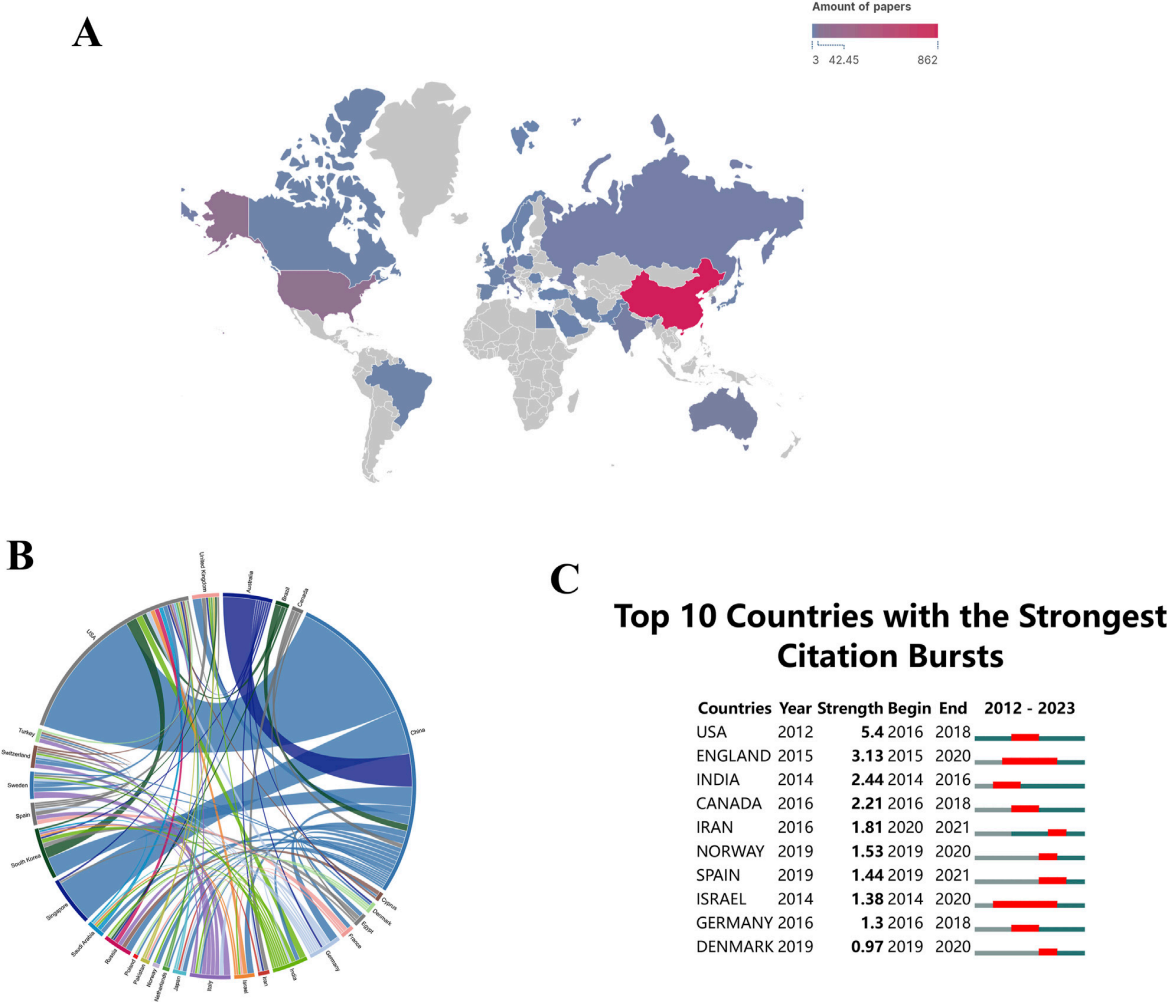
**(A)** Global distribution of research on “ferroptosis-nano drug delivery systems (NDDSs)”. **(B)** Chord diagrams showing international collaborations, with outer curves representing countries and line thickness indicating collaboration strength. **(C)** Research output on “ferroptosis-NDDSs” from the top 10 countries, highlighted in red to indicate increased production.

There are 60 countries/regions involved in the global research on “ferroptosis-based NDDSs”. Maps depicting national collaborations were generated for Figures 5A, B, utilizing a minimum threshold of three publications per country/region. Significantly, China is the leading contributor with 862 publications, comprising 82.09% of the total in this field. The United States and Australia, after China, contribute 10% (105 publications) and 0.38% (22 publications), respectively, to global “ferroptosis-based NDDS” research.

Countries and regions are represented by peripheral curve segments, each segment’s length reflecting the volume of publications for that country or region. Furthermore, the thickness of the connections between these nations signifies the degree of their collaborative efforts. During the study period, there was significant international collaboration. With a link strength of 60, China and the United States exhibited the most frequent academic collaborations (Figure 5B). The prominence of a country or region in this field is reflected in the number of publications (Joshi, 2014). In terms of academic influence, both China and the United States demonstrate substantial contributions to “ferroptosis-based NDDSs” research, indicating a strong collaboration ready to advance theoretical knowledge and address current field challenges (Dara et al., 2017).

Citation bursts are crucial in recognizing publications that have experienced significant increases in citations within a specified time frame (Cao et al., 2023a). These bursts reveal dynamic trends and orientations in research fields. Research areas of considerable scholarly interest can be identified through the analysis of rapidly cited publications. An illustration of the citation bursts for the top 10 nations is shown in Figure 5C, with red lines representing the magnitudes of each country’s bursts. During the period spanning from 2016 to 2018, there was a notable rise in publication citations for the United States (with a strength of 5.4), closely trailed by England (with a strength of 3.13). The strength of these bursts indicates that these countries/regions produced highly influential work during this period, which likely shaped subsequent research directions.

Research on “ferroptosis-based NDDSs” has rapidly expanded, with China emerging as the leading contributor. This dominance can be attributed to several factors: 1. Strategic National Initiatives: The Chinese government has prioritized biotechnology and nanotechnology within its broader innovation-driven development strategy. Initiatives like the “Made in China 2025”plan have directed substantial funding toward cutting-edge research areas, including NDDSs, fostering rapid advancements and high publication output. 2. Research Infrastructure and Collaboration: China has heavily invested in building world-class research infrastructure, attracting top talent, and fostering collaborations both domestically and internationally. The high publication volume is a testament to the efficiency of these efforts. The significant link strength of 60 between China and the United States, reflecting frequent academic collaborations, underscores the importance of international partnerships in advancing this field. 3. Government Policies Supporting Research: Policies incentivizing high-impact publications, international collaboration, and patent applications have propelled Chinese researchers to the forefront of this field. These policies have increased not only the quantity of research output but also its quality and impact.

However, the concentration of research output in China raises concerns about diversity and global representation in this field. A significant portion of the literature originating from a single country risks research homogeneity, where certain methodologies, theoretical approaches, and areas of focus become predominant. This could stifle innovation and hinder the development of alternative perspectives. Moreover, despite substantial international collaboration, the dominance of Chinese institutions may lead to imbalances in collaborative dynamics. Countries with fewer resources or less-developed research infrastructure may struggle to establish equal partnerships, potentially leading to a hierarchical structure in global research collaborations.

### 3.3 Institutional performance

**FIGURE 6:**
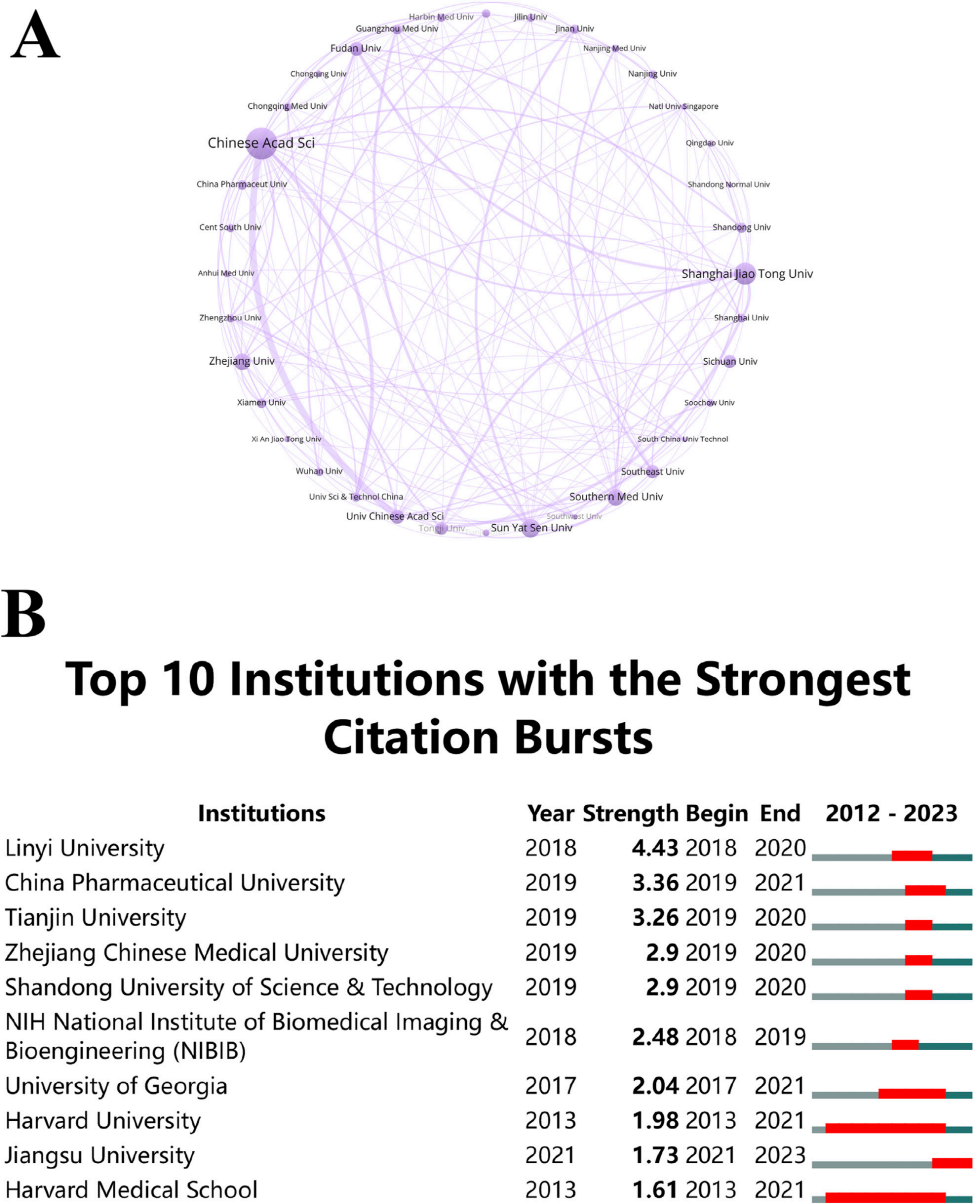
**(A)** Institutional co-occurrence analysis map. Spheres and text represent institutions, with lines indicating collaborations. Line thickness reflects collaboration strength, and circle size correlates with publication volume. **(B)** Citation bursts in the top 10 institutions, with red bars representing burst periods.

Researchers exploring potential collaborators and funding prospects can gain valuable insight by identifying high-performing institutions and examining their citation bursts. An ecosystem of thriving research is evidenced by the dynamic collaborative network between these institutions. Over the last 11 years, substantial advancements have occurred in the global research landscape related to “ferroptosis-based NDDSs”, engaging with over 1,000 entities. Notably, the Chinese Academy of Sciences emerges as the predominant contributor, representing about 8.57% (90 publications) of the overall research output. Shanghai Jiao Tong University and Sun Yat-sen University follow closely, contributing 5.71% (60 publications) and 4.67% (49 publications) respectively. Establishing a collaboration network among these institutions, as depicted in Figure 6A, involved a prerequisite of at least 12 publications for inclusion. In the network, each node (a sphere-text combination) represents an institution, and the lines indicate collaborative instances. The degree of cooperation is reflected in the thickness of the lines, and the institution’s publication count correlates with the size of the circles. This analysis revealed a dynamic collaborative network, significantly involving the Chinese Academy of Sciences and the University of Chinese Academy of Sciences. The thriving research ecosystem within Chinese institutions, marked by active collaboration and substantial publication output, is pivotal in advancing “ferroptosis-based NDDSs”. Collaborating with these leading institutions allows researchers from other regions to access extensive expertise and resources, accelerating the development of new therapies and technologies.

CiteSpace analysis (Figure 6B) identified institutions experiencing notable citation bursts. Harvard University and Harvard Medical School, from 2013 to 2021, underwent a substantial surge in citations, followed by a decline in the subsequent 2 years. In contrast, the citation burst of Jiangsu University during 2021–2023 signifies a change in emphasis and a delay in research productivity. These shifts in citation bursts underscore the dynamic nature of research trends, with different institutions leading at different times. This underscores the need to stay attuned to these trends when seeking collaborators or funding, as emerging leaders may introduce fresh ideas and innovative approaches to the field.

The dominance of the Chinese Academy of Sciences and other top Chinese universities is not coincidental but the result of strategic investments in research infrastructure, talent development, and international collaborations. These institutions have established themselves as global leaders, attracting significant funding and producing high-impact research. The Chinese government has implemented policies aimed at fostering innovation and scientific excellence. Programs like the “Double First Class” initiative, aimed at developing world-class universities and disciplines, have provided substantial support to top institutions like the Chinese Academy of Sciences, Shanghai Jiao Tong University, and Sun Yat-sen University. These policies have encouraged cutting-edge research, including in emerging fields like NDDSs. Although Chinese institutions dominate the research landscape, they maintain strong international collaborations, notably with Western institutions like Harvard University and Harvard Medical School.

The concentration of research within a few dominant institutions could create an echo chamber, where certain theories, methodologies, and approaches are repeatedly reinforced, marginalizing alternative perspectives. This could hinder the development of novel ideas and reduce the field’s adaptability to new challenges. To mitigate the risks of concentrated authorship, it is essential to encourage more diverse contributions from institutions across different regions. This involves promoting collaborative research with a broader range of institutions, supporting open-access publishing to democratize knowledge dissemination, and incentivizing interdisciplinary approaches to introduce fresh perspectives from related fields.

### 3.4 Author contributions

**FIGURE 7:**
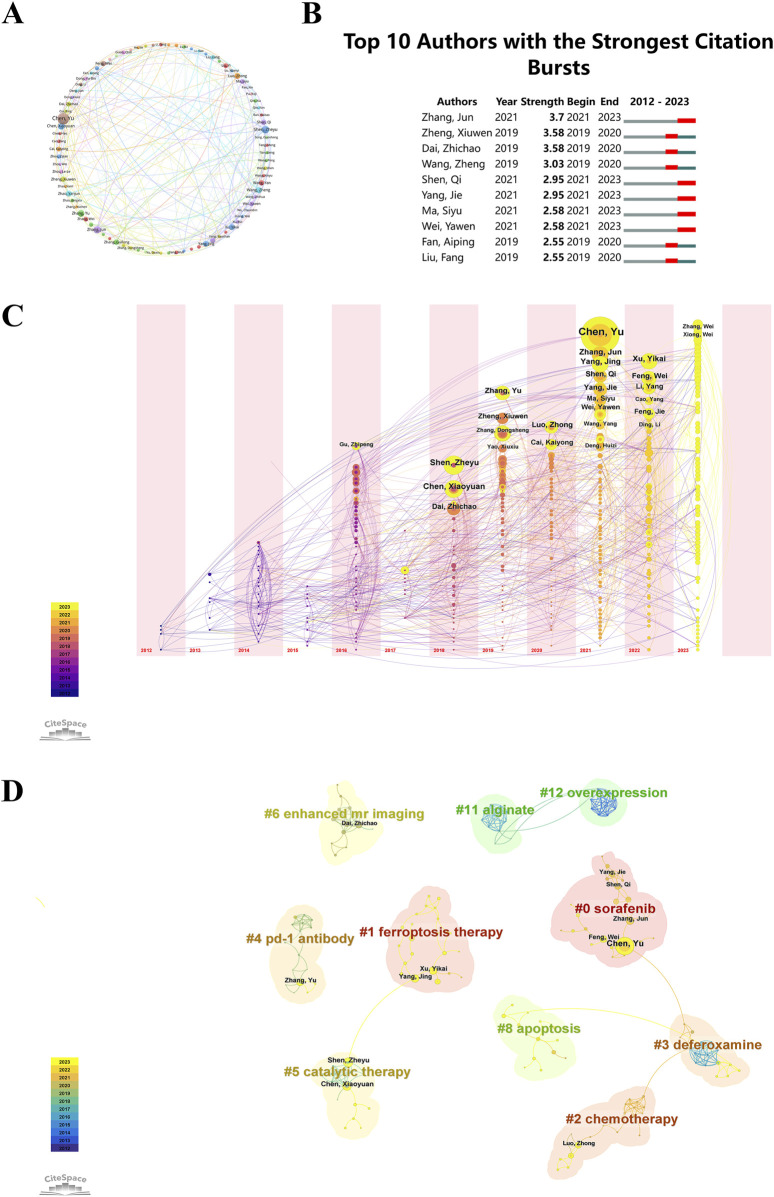
**(A)** Co-occurrence map of authors, with circles and labels forming nodes. Different colors represent distinct clusters. **(B)** Top 10 authors with the strongest citation bursts in “ferroptosis-nano drug delivery systems” publications. **(C)** Temporal overlay of the author’s collaborative network. Each sphere represents an author, with the size of overlapping spheres indicating total authored articles. Purple-red indicates earlier publications, yellow recent ones, and overlapping colors show multi-year publishing. These create a ring-like pattern, with connections showing collaborative occurrences. **(D)** Cluster analysis of authors, where circle size correlates with publication volume. Purple denotes earlier publications, yellow later ones, and overlapping colors represent annual publications. Lines between circles indicate co-authorship.

Researchers can pinpoint key figures in the field by identifying productive contributors and analyzing their collaborative networks. Authors who frequently receive citations and have a long history of publishing offer important perspectives that influence the direction of future research efforts. Among the “ferroptosis-based NDDSs”, 6,244 authors were notable contributors. Ten authors contributed ten or more papers, demonstrating impressive productivity. Employing VOSviewer software for an in-depth exploration of co-authorship networks, we generated visualization maps, setting a threshold of a minimum of five publications per author. Each author’s publication number is represented by the dimensions of the circle in these visualizations. Clusters of authors are indicated by the color schemes, and the strength of collaboration is depicted by the thickness of the connecting lines. Notably, the publication threshold was surpassed by 85 authors, showcasing the most substantial collaborative partnership between Wang Zhen and Zhao Yanjun, as illustrated in Figure 7A. Additionally, the pivotal roles of Chen Yu, Shen Zheyu, and Zhang Jun in advancing research on “ferroptosis-based NDDSs” were underscored, highlighting their significant contributions to this scientific domain.

During a defined period, citation burst analysis measures the number of citations an author receives in a particular research domain. In Figure 7B, the top ten authors in the field of “ferroptosis-based NDDSs” received the highest citation counts. Leading the pack is Zhang Jun, displaying a citation burst strength of 3.7, closely followed by Zheng Xiuwen and Dai Zhichao. Noteworthy is the increased publication activity over the past 3 years by authors such as Zhang Jun, Shen Qi, Yang Jie, Ma Siyu, and Wei Yawen, indicating their heightened research efforts in this field. These authors, due to their high citation frequencies, make significant contributions to the understanding of “ferroptosis-based NDDSs” and offer a reference framework that may potentially influence future research directions in this area.

A simultaneous analysis of nodes was conducted for the same time periods. This analysis is depicted in a graph, with each sphere representing an individual author. Sphere size corresponds to the author’s total publication volume. Each node’s color represents the publication timeline: purple for earlier works and yellow for more recent ones. Combined colors on a node represent the author’s publications in that year. Multiple overlapping colors on a node create an ‘annual wheel’, signifying consistent and prolific publishing (Figure 7C). Chen Yu (n = 23, 2.19%), Shen Zheyu (n = 14, 1.33%), and Zhang Jun (n = 13, 1.24%) were notable for their high productivity and consistent publication records over time.

CiteSpace evaluates network integrity and clustering clarity using Modularity (*Q* value) and Mean Silhouette (*S* value). A value above 0.5 for *S* indicates clear clustering, while a *Q* value above 0.3 suggests robust clustering. The study reports a cluster modularity value (*Q*) of 0.9647 and a mean silhouette value (*S*) of 0.9914, indicating highly significant and accurate keyword clustering. In Figure 7D, authors are categorized into 10 groups and labeled with keywords such as: #0 sorafenib, #1 ferroptosis therapy, #2 chemotherapy, #3 deferoxamine, #4 PD-1 antibody, #5 catalytic therapy, #6 enhanced MR imaging, #8 apoptosis, #11 alginate, and #12 overexpression.

The prominence of Chinese researchers in this field is partly due to national research policies that emphasize collaboration and innovation. China’s strategic focus on scientific research and technological development has created an environment that strongly encourages collaboration between institutions and researchers. Programs that fund collaborative research and incentivize publications in high-impact journals have significantly boosted the visibility and impact of Chinese researchers. However, the concentration of authorship among a few prolific researchers, especially in Chinese institutions, has both positive and negative consequences. On the one hand, this results in a robust body of work with consistent quality and direction. On the other hand, this could stifle idea diversity, which is crucial for the research field’s long-term vitality and adaptability. To promote a more diverse and inclusive research environment, it is essential to encourage contributions from a wider range of researchers and institutions. This may include promoting interdisciplinary research, supporting emerging scholars, and ensuring that publication venues are accessible to researchers from diverse backgrounds and regions.

### 3.5 Analysis of high-contributing journals

**FIGURE 8:**
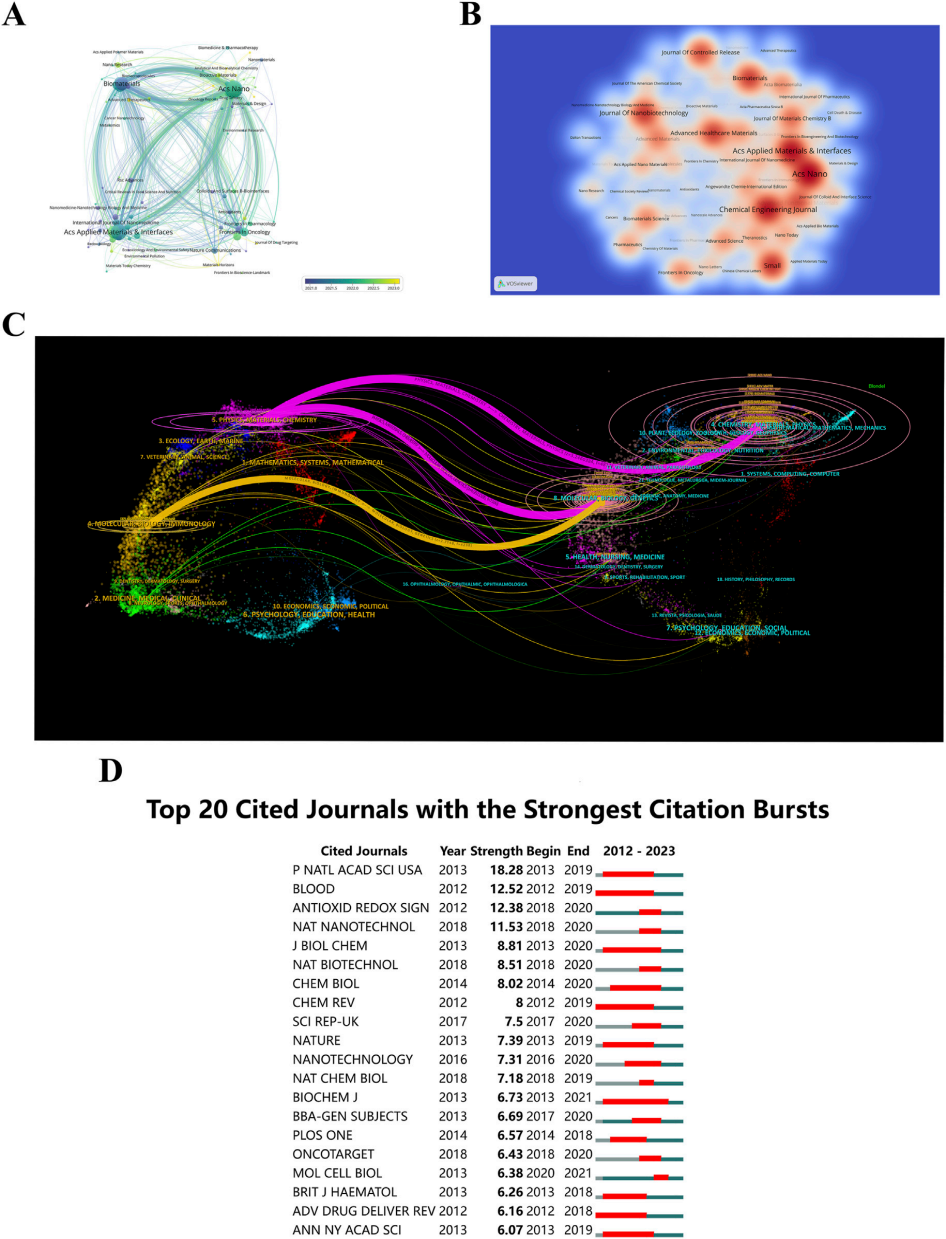
**(A)** Journal distribution by average publication year (blue: earlier, yellow: later). Each circle and label form a node, with circle size reflecting keyword frequency. The color gradient indicates the average year of occurrence. **(B)** Density visualization of journal citations, with color intensity reflecting publication volume. **(C)** Dual-map overlay of journals related to “ferroptosis-nano drug delivery systems”, where each point represents a journal and curves show citation connections. These trajectories reveal interdisciplinary relationships and the evolution of citations. **(D)** Top 20 journals with the highest citation bursts.

Understanding the journals that publish research on “ferroptosis-based NDDSs”, their years of emergence, and citation dynamics helps researchers select appropriate publication venues and stay updated on the latest developments in the field. The mean year of establishment of journals of various colors is shown in Figure 8A. A circle’s color indicates the mean year of publication, while its size and label indicate frequency. It is evident that *ACS Materials Letters* and *Advanced Therapeutics*, characterized by their yellow coloration, are emerging journals. A visual representation of journal publication data reveals the presence of articles related to “ferroptosis-based NDDSs” across 276 journals. With a minimum threshold of four documents per journal, a thermodynamic chart depicts document distribution among these journals, with the color intensity reflecting the number of papers published in each journal (Figure 8B). *ACS Nano* leads in document count with 50 publications (4.76%), succeeded by *ACS Applied Materials & Interfaces* with 44 documents (4.19%), and *Chemical Engineering Journal* with 42 documents (4%).

An insightful tool, the dual-map overlay of journals displays the interdisciplinary distribution of journals, how citation trajectories have evolved, and how scientific research centers have shifted (Yuan et al., 2023). Labels on the map delineate the diverse study areas encompassed by all journals. Citations are situated on the left, while references are positioned on the right. Interactions are visually represented by reference paths, vividly colored lines originating from the citation map and terminating there. There is strong correlation between the width of these pathways and the frequency of citations on a *z*-score basis (Pei et al., 2022). Figure 8C illustrates the linkage of “ferroptosis-based NDDSs” research to six main categories: molecular biology, immunology, genetics, physics, materials, and chemistry. Furthermore, illustrated in Figure 8D, are the leading 20 journals exhibiting the highest citation rates for publications related to “ferroptosis-based NDDSs”.

### 3.6 Co-cited references

**FIGURE 9:**
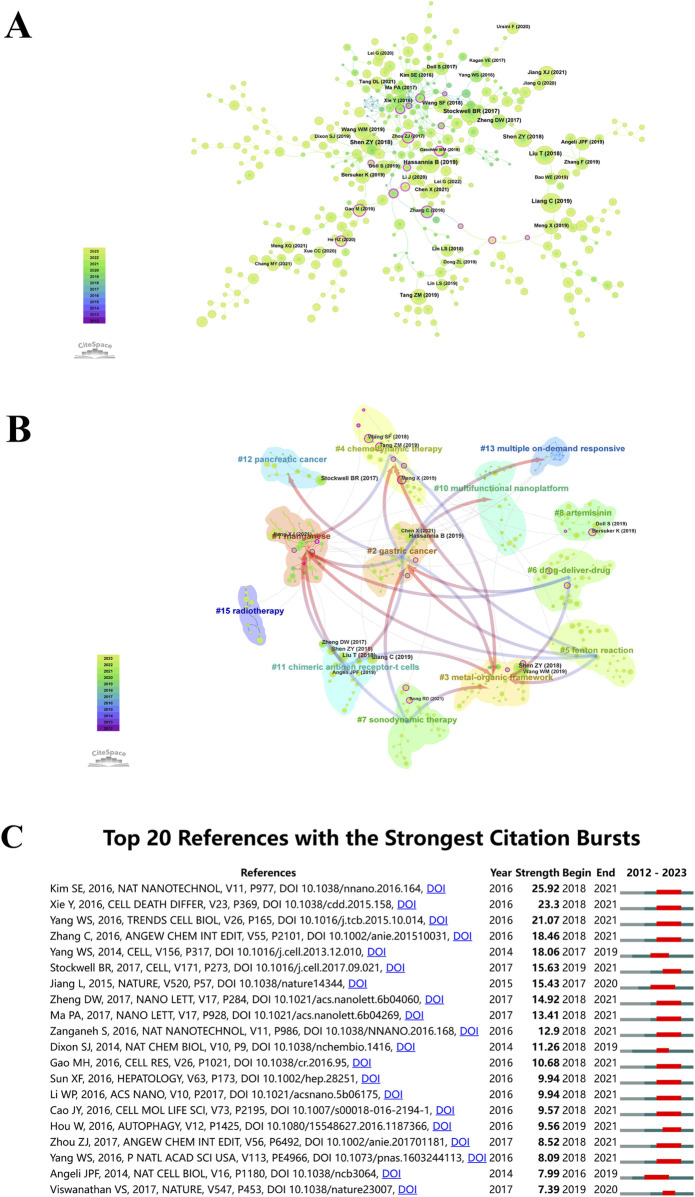
**(A)** Co-citation analysis for “ferroptosis-nano drug delivery systems”. Circle sizes are proportional to citation counts, with purple indicating earlier citations, green for later ones, and overlapping colors showing citations by year. Lines connect co-cited literature, and magenta nodes indicate key nodes with centrality greater than 0.1. **(B)** Co-cited literature network map with similar annotations. **(C)** Top 20 references with the highest citation bursts.

An illustration of the co-citation network of literature on “ferroptosis-based NDDSs” is presented in Figure 9A, analyzed using CiteSpace between 1 January 2012, and 30 November 2023. This depiction consolidates the sphere sizes across yearly rings, proportional to their co-citation frequency. Earlier citations are shown in purple, while more recent citations are shown in yellow-green. The overlapping colors on the spheres indicate sustained citations over the specified period. Co-citation relationships among various literature pieces are illustrated by the interconnections between spheres. Those nodes in the network with magenta highlights have a centrality greater than 0.1, which defines them as pivotal nodes in the network. The paper titled ‘Ferroptosis: A Regulated Cell Death Nexus Linking Metabolism, Redox Biology, and Disease’, authored by Brent Stockwell et al. and published in *Cell* in 2017, obtained the maximum co-citation count (n = 16) (Stockwell et al., 2017). Following closely, the 2018 *Advanced Materials* paper ‘Emerging Strategies of Cancer Therapy Based on Ferroptosis’ by Shen Zheyu et al. obtained 155 co-citations (Shen et al., 2018).

CiteSpace uses Modularity (*Q* value) and Mean Silhouette (*S* value) metrics to evaluate network structures and clustering clarity. A *Q* value over 0.3 suggests significant clustering, and an *S* value above 0.5 indicates effective clustering. The conducted analysis yielded *Q* = 0.8729 and *S* = 0.9702, confirming the presence of robust clustering structures. It is clear from this outcome that the clustering method applied is reliable. The analysis identified 13 distinct clusters, including manganese (#1), gastric cancer (#2), metal-organic framework (#3), chemodynamic therapy (#4), Fenton reaction (#5), drug-delivery-drug (#6), sonodynamic therapy (#7), artemisinin (#8), multifunctional nanoplatform (#10), chimeric antigen receptor-T cells (#11), pancreatic cancer (#12), multiple on-demand responsiveness (#13), and radiotherapy (#15), as illustrated in Figure 9B.

Utilizing CiteSpace’s analytical features, citation bursts were identified to highlight studies receiving significant scholarly attention in “ferroptosis-based NDDSs”. Figure 9C illustrates how the top 20 references were marked by significant citation bursts due to their profound impact. In the years following 2016, the field witnessed an upswing in citations, with several co-cited references accumulating substantial citation counts. Researchers studying “ferroptosis-based NDDSs” will continue to benefit from research on this topic. The year 2018 stands out, with 70% (14 out of 20) of these references experiencing citation bursts, marking it as the year with the highest frequency. Following closely are 2019 and 2017, constituting 15% (3 out of 20) and 10% (2 out of 20) of the bursts, respectively. Notably, ‘Ultrasmall nanoparticles induce ferroptosis in nutrient-deprived cancer cells and suppress tumor growth,’ originally published in *Nature Nanotechnology* (Kim et al., 2016). The works of Xie Y et al. (Xie et al., 2016), and Yang et al. (Yang and Stockwell, 2016) closely followed.

### 3.7 Keyword analysis

**FIGURE 10:**
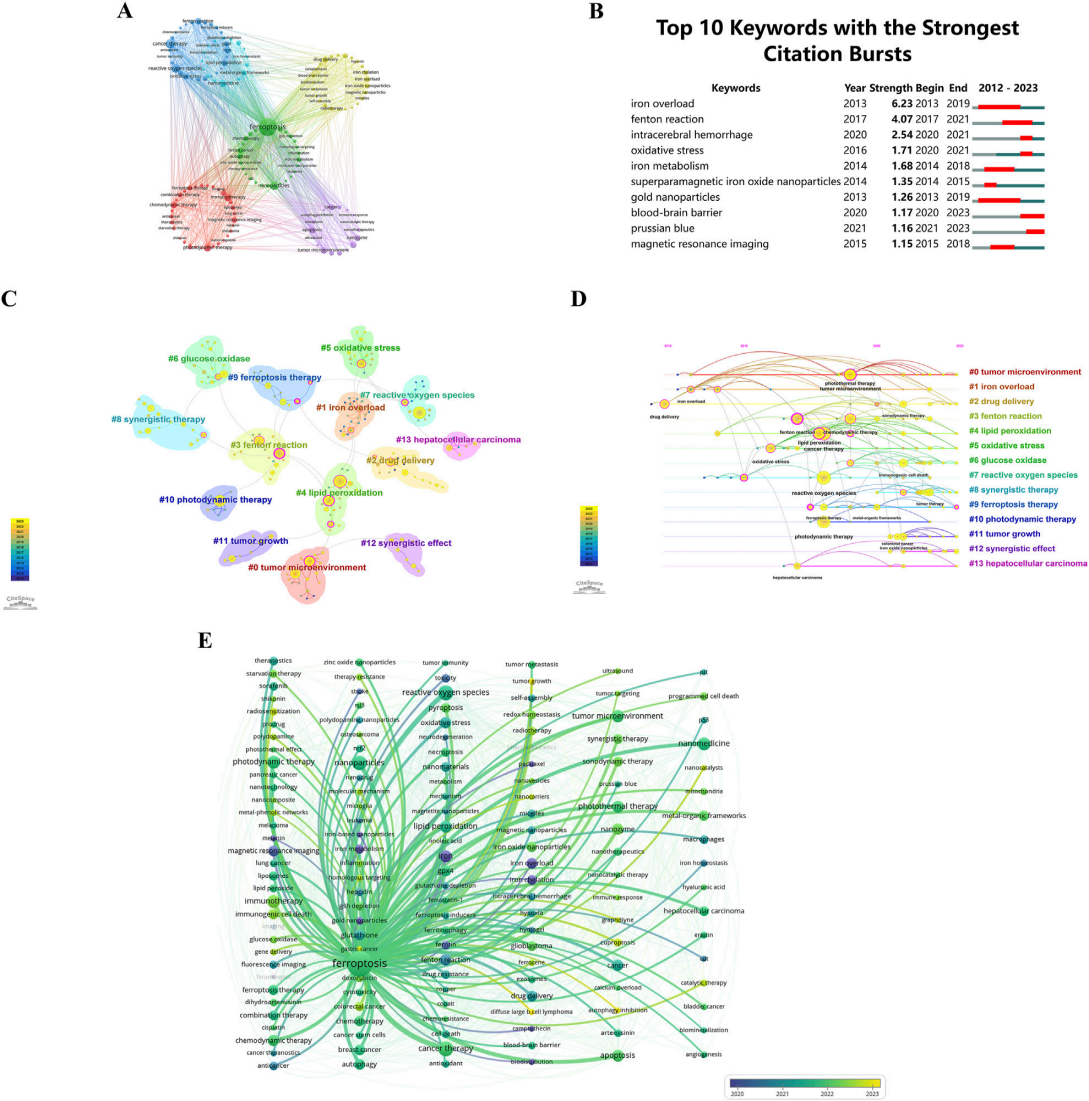
**(A)** Keyword clustering visualization. Circles and labels form nodes, with circle size correlating with keyword frequency. Line thickness reflects the strength of keyword relationships. Different colors indicate distinct research clusters. Keywords with high similarity are grouped together using co-occurrence clustering algorithms. **(B)** Top 10 keywords with the strongest citation bursts by CiteSpace. **(C)** Keyword frequency cluster analysis. **(D)** Temporal trends in keyword co-occurrence. Circle sizes reflect keyword frequency, and lines show co-occurrence. Purple represents earlier keywords, yellow later ones, and overlapping colors indicate appearance across years. Magenta nodes are central, acting as hubs. Keywords are arranged by first appearance, progressing rightward. **(E)** Temporal frequency spectrum of “ferroptosis-nano drug delivery systems” keywords. Line thickness reflects correlation strength, with blue for earlier, classical keywords and yellow for recent, emerging trends.

Clustering and burst analysis of keywords provide a deeper apprehending of research themes and burgeoning topics. Researchers can use this information to identify hotspots, explore new directions, and ensure their work aligns with current trends (Cao et al., 2023a). A visual map (Figure 10A) has been generated using co-occurrence cluster analysis on keywords. In this map, nodes, depicted as labeled circles, were utilized. Circle sizes directly correlate with keyword frequency, while relationship strength is depicted by the connecting lines’ thickness. Color-coding has been used to categorize the nodes into distinct clusters, with each color representing a distinctive research focus. In total, the analysis has successfully identified five clusters.

Keyword bursts, characterized by a significant increase in citations within a specified timeframe, are prominently illustrated in Figure 10B. This depiction shows that 30% of the keywords (3 out of 10) had their initial citation burst in 2020, with 2013 and 2014 each contributing to 20% of the bursts (2 out of 10). Significantly, 50% of these keywords have observed an upsurge in citation rates during the preceding 3 years, emphasizing the sustained and growing interest in particular research domains. With a burstness value of 6.23, “iron overload” registered the largest burst, followed by “Fenton reaction” with a burstness value of 4.07 and “intracerebral hemorrhage” with a burstness value of 2.54. There are substantial implications for researchers within these fields as a result of these findings.

CiteSpace uses Modularity (*Q* value) and Mean Silhouette (*S* value) metrics to assess network structures and clustering clarity. Modularity values exceeding 0.3 indicate significant clustering, while Mean Silhouette values above 0.5 indicate effective clustering. The analysis yielded a *Q* value of 0.8664 and an *S* value of 0.9759, validating the substantial and clear clustering within the network. These results emphasize the effectiveness and reliability of the citation clustering methodology employed in this study. The analysis identified 14 distinct clusters, labeled as follows: #0 tumor microenvironment, #1 iron overload, #2 drug delivery, #3 Fenton reaction, #4 lipid peroxidation, #5 oxidative stress, #6 glucose oxidase, #7 reactive oxygen species, #8 synergistic therapy, #9 ferroptosis therapy, #10 photodynamic therapy, #11 tumor growth, #12 synergistic effect, and #13 hepatocellular carcinoma. These clusters are illustrated in Figure 10C.

In Figure 10D, a temporal examination of keyword frequency clustering in hotspots is depicted. Circle sizes reflect the frequency of each keyword. There is a correlation between keywords based on their interconnections. There is significance to the color code: purple indicates earlier keyword emergence, yellow signifies more recent appearances, and overlap between these two colors indicates keywords occurring throughout the respective time period. A magenta node demonstrates a node’s pivotal role as a hub by highlighting keywords that are highly centralized. Horizontally organized nodes belong to the same cluster, with the timeline oriented from the top for the initial occurrence of a keyword, progressing chronologically to the right. With this visualization, you can determine the relevance of each cluster based on the quantity of keywords within it. Keywords in each cluster are also categorized by their temporal span. Keywords are grouped into 14 distinct categories: #0 tumor microenvironment, #1 iron overload, #2 drug delivery, #3 Fenton reaction, #4 lipid peroxidation, #5 oxidative stress, #6 glucose oxidase, #7 reactive oxygen species, #8 synergistic therapy, #9 ferroptosis therapy, #10 photodynamic therapy, #11 tumor growth, #12 synergistic effect, #13 hepatocellular carcinoma. These clusters, encapsulating the primary themes and hotspots in the research domain, reflect scientific advancements, therapeutic potentials, and clinical applicability.

Figure 10E presents a chronological keyword spectrum related to ‘ferroptosis’, where line thickness indicates the association’s intensity. Keywords in blue represent earlier appearances, indicating their foundational status, while those in yellow denote recent developments, suggesting emerging research directions. This spectrum effectively illustrates the evolution of research themes, key concepts, and interrelationships over time, offering a comprehensive perspective on the knowledge landscape and research endeavors within this front, enabling deeper insights into current research trends.

### 3.8 Related diseases

**FIGURE 11:**
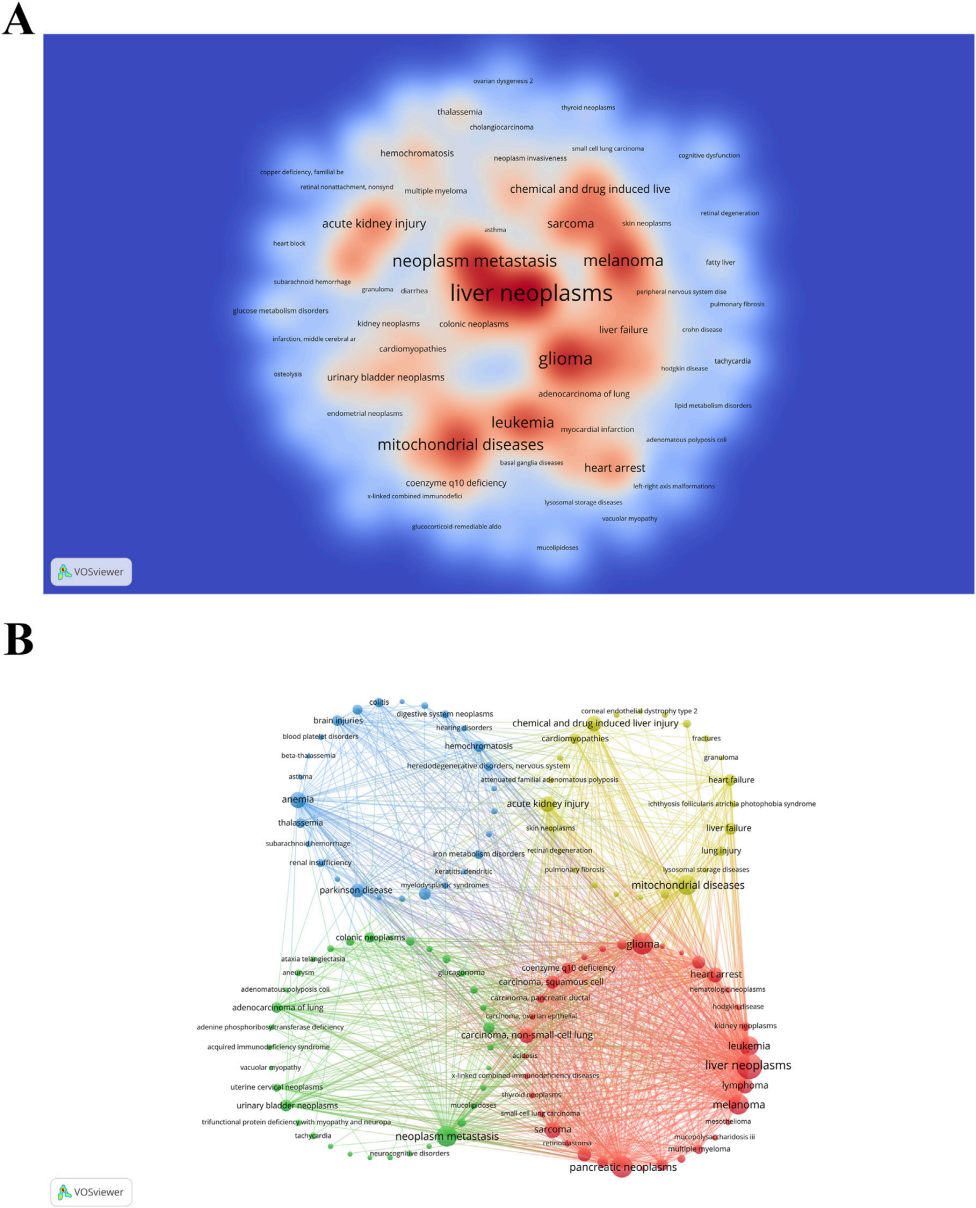
**(A)** Density visualization map of associated diseases, with color intensity directly proportional to disease frequency. **(B)** Disease clustering analysis chart. Circles and labels form nodes, with circle size correlating with disease frequency, and line thickness indicating the strength of disease relationships. Different colors represent distinct disease clusters.

Identifying the diseases most closely linked with research on “ferroptosis-based NDDSs” can assist researchers in targeting specific medical conditions for drug delivery applications. Utilizing the Citexs Data Platform, 749 diseases were identified across 1,050 articles, with a minimum occurrence of five articles for each disease. A heatmap, generated through VOSviewer, illustrates the research landscape pertaining to “ferroptosis-based NDDSs” and showcases diseases meeting the aforementioned criterion (Figure 11A). The five most frequently mentioned diseases are malignant tumors, including liver neoplasms, pancreatic neoplasms, glioma, neoplasm metastasis, and melanoma. Employing VOSviewer, a co-occurrence cluster analysis was undertaken (Figure 11B), necessitating a minimum occurrence threshold of five instances for each disease. Within this graphical representation, nodes, symbolized by circles and labels, are proportionate to the frequency of each disease. The lines connecting these circles vary in thickness, reflecting the strength of relationships among diseases. Additionally, nodes of diverse colors amalgamate to create discernible clusters, with each color denoting a specific category of disease clusters.

## 4 Discussion

Keeping up with industry developments and understanding the latest research findings has become increasingly challenging in the age of information proliferation. To disseminate the contemporary state of research on a global scale, specifically focusing on “ferroptosis-based NDDSs” from 2012 to 2023, we have employed bibliometric analysis. Research frameworks within specific research domains can be elucidated and administered using this methodology.

### 4.1 Overview of salient results

Over the past 11 years, an increasingly substantial body of research has elucidated the pivotal role of ferroptosis in a spectrum of pathological conditions, encompassing oncological disorders, neurodegenerative ailments, tissue and organ injuries, as well as various inflammatory diseases, thus substantiating its status as a *bona fide* therapeutic target (Wang X. et al., 2023). While nanomaterials designed to target ferroptosis have not yet garnered clinical approval, they have exhibited significant promise (Sun et al., 2023; Liu Q. et al., 2023; Luo et al., 2021). There is a direct correlation between the number of relevant research articles originating from a particular geographic region, the economic prowess, and the level of scientific research. The predominant top 10 countries/regions and top 10 institutions are mainly situated in Europe, North America, and Asia. With the exception of China, India, and Russia, all others are categorized as developed nations. A distinct correlation emerges between academic output and economic prowess, signifying that advanced nations or esteemed institutions wield considerable advantage and influence.

China and the United States, being the two largest global economies, also demonstrate the highest dedication and intensity when it comes to collaborative scientific efforts. Their combined publication count constitutes a substantial 92.09% of the total worldwide, underscoring their significant impact on the field. Insufficient investment, low industrial confidence, limited incorporation of nanotechnology, and an absence of readiness and strategic planning for societal contingencies pose inherent barriers for less developed countries/regions concerning their academic, industrial, and economic competitiveness in the field. Research and development in nanomedicine industry, as well as commercialization, are hindered by these obstacles. As global industrialization progresses and healthcare facilities expand, an escalation in transnational and cross-institutional collaborations is inevitable between developed and less developed countries/regions. Foreseen are valuable opportunities for the latter through these collaborations, combined with the transfer of nanoproduct authorization (Wang et al., 2023c).

International collaboration has occupied a significant place in advancing the field of “ferroptosis-based NDDSs” in several significant ways. Firstly, it promotes academic synergy, harnessing the unique expertise and strengths of diverse nations, like China and the United States, across various subdomains. Collaborative endeavors facilitate the sharing of resources and the realization of mutual benefits. For example, significant advancements in a specific scientific field could be achieved by one nation, whereas another excels in fundamental research and the application of technology. Cooperative efforts across borders facilitate the exchange of knowledge and technology transfer. Furthermore, international cooperation nurtures shared research interests. Collaborative endeavors among nations with shared research objectives in specific domains can enhance their research capabilities and productivity. This collaborative approach involves engaging in joint research initiatives, forming cross-border scientific teams, and co-authoring publications. Additionally, international collaboration provides crucial financial and infrastructure support. Diverse nations collaborating often bring complementary resources to the table, combining funding from one party with access to research facilities or laboratory equipment from another, thereby accelerating advancements in the field of “ferroptosis-based NDDSs”. Ultimately, participating in international collaborations elevates the global standing and recognition of the involved countries, contributing to an enhanced reputation within the international academic community. Such collaborations frequently pave the way for additional opportunities for cooperation, access to resources, and increased influence.

Examining the relationship analysis depicted in Figure 6A reveals that the leading ten institutions in the realm of “ferroptosis-based NDDSs” predominantly originate from China. This trend is further underscored by the publication highlight chart (Figure 6B). In summary, these findings underscore China’s global dominance, encompassing both the overall publication count and the pace of publications. Significantly, most of these institutions demonstrate a preference for collaborations within the country rather than on an international scale, a tendency shaped by the prevalent collaboration patterns within China. In order to promote extensive international knowledge exchange, share resources, and integrate expertise, it is crucial to prioritize future collaborations between Chinese institutions and their international counterparts. Understanding the research institution landscape and their collaborative tendencies is essential for advancing the field of “ferroptosis-based NDDSs”. Encouraging innovation and ultimately enhancing human wellbeing are critical aspects of fostering sustained research efforts, nurturing international partnerships, and leveraging the capabilities of renowned institutions.

Examining the co-citation analysis chart (Figure 9A), it becomes apparent that the work authored by Brent Stockwell in 2017 has garnered the highest count of co-citations within the present investigation. Renowned as a trailblazer in ferroptosis research, Brent Stockwell’s contributions are highly regarded in the global academic community. Throughout the past 11 years, he has spearheaded advancements in ferroptosis research, assuming a leading role in shaping ensuing academic investigations. His consistent publication of research achievements and remarkable achievements during the early stages of this field accentuates the importance of ongoing contributions by distinguished investigators in upholding the advancement and vitality of the discipline.

The analysis of keyword clustering (Figures 10C, D) reveals the predominant research areas encompassing the tumor microenvironment (TME), iron overload, Fenton reaction, synergistic and photodynamic therapy (PDT), among others. Nonetheless, persisting challenges demand immediate resolution. Specifically concerning the TME, there is an exigent requirement for an enhanced comprehension of the interplay between ferroptosis and immune responses to facilitate the development of more precisely targeted nanomaterials (Luo et al., 2021). Within the context of the relationship between ferroptosis and antitumor immunity, a dearth of discoveries necessitates further investigations aimed at refining our understanding of ferroptosis within immune and cancer cells, with the aim of elucidating their distinctions (Xu et al., 2021). The pivotal advancement of nanomaterials capable of selectively targeting cells based on intercellular disparities emerges as a critical determinant in promoting both ferroptosis and immunotherapy. Addressing intracellular iron overload and Fenton production, it is imperative to acknowledge that a weak intracellular acidic milieu results in diminished rates of reactive oxygen species (ROS) generation, contingent upon hydrogen peroxide production (Yao et al., 2023). In the context of synergistic therapy, the precise effects of individual systems, their regulation, interactions among multiple systems, and the potential for cumulative systems to induce prolonged human toxicity require further comprehensive experimental validation. Concerning PDT, notwithstanding the multifaceted roles of ROS generated during ferroptosis and PDT treatments, encompassing DNA damage, drug resistance, cell death, and genetic resilience, and their pivotal involvement in synergistic outcomes (Wang H. et al., 2023), it is imperative to recognize their proclivity to promote cellular proliferation and viability in specific instances (Wang et al., 2022c). Furthermore, the hypoxic TME imposes constraints on the antitumor efficacy of PDT, predicated upon external stimuli (Liu Q. et al., 2023). Thus, an imperative mandate exists for the comprehensive exploration of the synergistic mechanisms underpinning ferroptosis and PDT.

The prioritization of cancers like liver neoplasms, pancreatic neoplasms, glioma, neoplasm metastasis, and melanoma in “ferroptosis-based NDDSs” research reflects a strategic focus on disease prevalence, the relevance of ferroptosis to these tumors, and the feasibility of using NDDSs in these cases (Figure 11A). Liver, pancreatic neoplasms, gliomas, and melanomas are particularly challenging to treat, with poor prognoses and high mortality rates. For instance, liver cancer is the sixth most common cancer worldwide and the third leading cause of cancer-related deaths (Sung et al., 2021). Pancreatic cancer also has notoriously low survival rates, with most cases diagnosed at an advanced stage, rendering conventional treatments largely ineffective (Pereira et al., 2020). Gliomas, especially glioblastoma multiforme, are the most aggressive primary malignancy of the brain, with limited treatment options and a median survival of 12–15 months (Cloughesy et al., 2014). The high prevalence and poor outcomes of these cancers highlight the urgent need for innovative treatment strategies. Prioritizing these cancers allows researchers to address significant unmet clinical needs, offering hope for improved survival rates and better patient quality of life.

Ferroptosis is inhibited in diverse cancer types and functions as a dynamic tumor suppressor in cancer development, indicating that the regulation of ferroptosis can be utilized as an interventional target for tumor treatment (Wang H. et al., 2021). For example, in glioma cells, when the availability of intracellular glucose decreases, glioma cells exhibit a high dependence on glutamine, and by inhibiting the formation of system Xc^−^, GSH cannot be adequately synthesized and amino acid metabolism is imbalanced, leading to ferroptosis can be achieved in the treatment of glioma (Yao et al., 2023). Melanoma cells, for instance, often develop resistance to traditional therapies like B-Raf proto-oncogene serine/threonine-kinase (BRAF) inhibitors, but ferroptosis offers a novel mechanism to overcome this resistance (Ta et al., 2023; Khorsandi et al., 2023). The specific relevance of ferroptosis to these cancers strongly justifies focusing on them in “ferroptosis-based NDDSs” research. Targeting the ferroptosis pathway allows researchers to exploit these cancers’ inherent vulnerabilities, potentially leading to more effective and targeted therapies.

The feasibility of NDDSs in treating these cancers is also crucial in their prioritization. NDDSs provide benefits like improved drug solubility, controlled release, and precise tumor targeting, reducing off-target effects and toxicity (Wang et al., 2022c). In liver and pancreatic cancers, where tumors are dense and located in challenging anatomical regions, NDDSs can enhance drug penetration and retention within the TME (Yu et al., 2024; Jia et al., 2021). In gliomas, protected by the blood-brain barrier, NDDSs can be engineered to cross this barrier, delivering ferroptosis-inducing agents directly to tumor cells (Cui et al., 2022; Song et al., 2024). Melanoma, as an external and highly vascularized tumor, is also well-suited for nano-delivery, where targeted approaches minimize systemic exposure and enhance drug concentration at the tumor site (Khan et al., 2022; Liu et al., 2018). The feasibility of NDDSs in these cancers ensures that the theoretical benefits of ferroptosis induction can be translated into practical, clinically viable treatments (Liu Q. et al., 2023; Zafar et al., 2021).

### 4.2 Current state-of-the-art approaches

#### 4.2.1 Triggering fenton reaction

**FIGURE 12:**
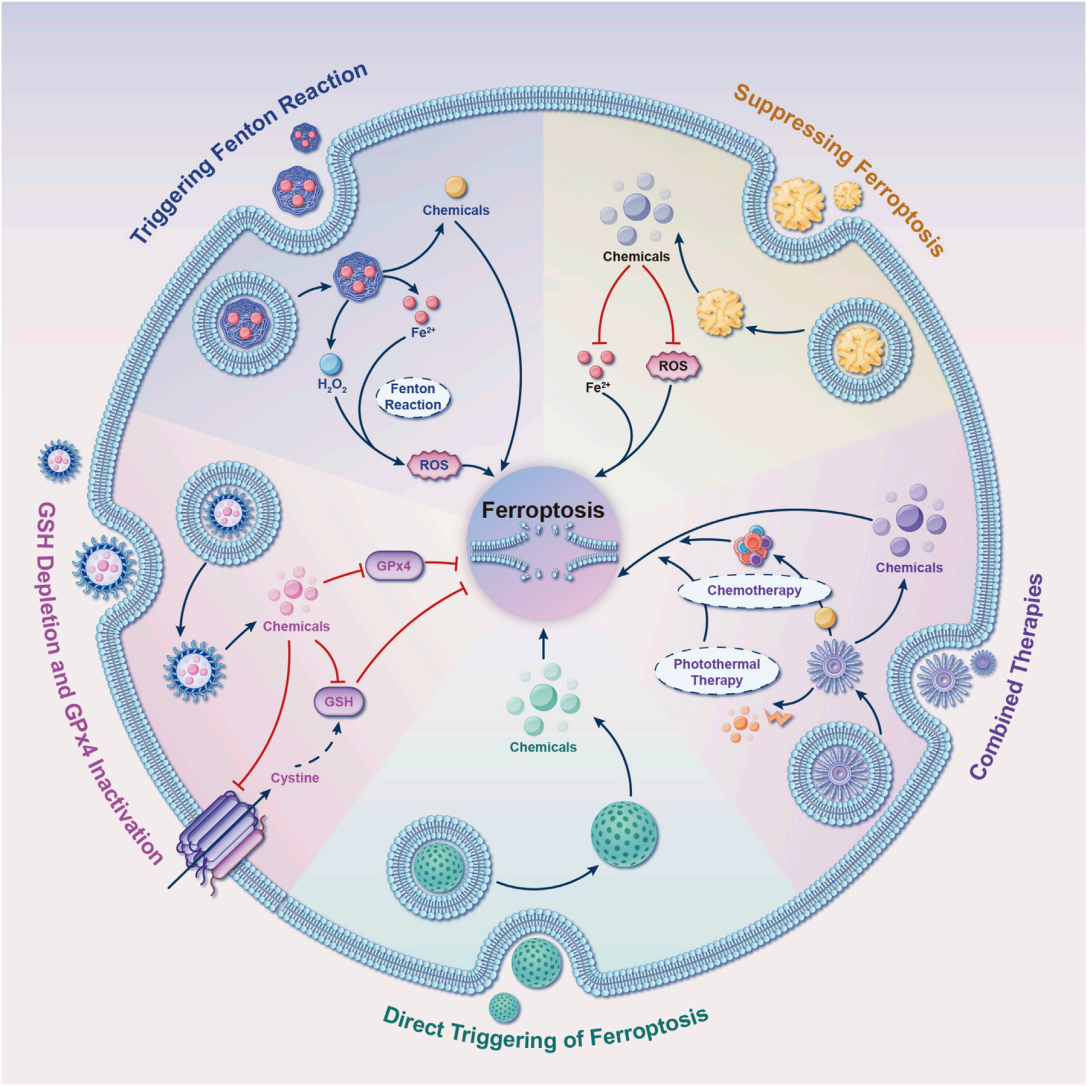
Five tactics for developing nano drug delivery systems (NDDSs) targeting ferroptosis. These strategies guide the design of NDDSs to modulate ferroptosis. They include the incorporation of compounds into NDDSs that either directly modulate ferroptosis or affect its regulatory signaling pathways. Abbreviations: GSH, glutathione; GPx4, glutathione peroxidase 4; ROS, reactive oxygen species.

Several NDDSs have been formulated to amplify the occurrence of ferroptosis, selectively triggering the Fenton reaction within cancerous cells (Figure 12). Zhang et al. reported the synthesis of amorphous iron nanoparticles (AFeNPs) (Zhang et al., 2016). AFeNPs initiated Fenton reactions within tumors by exploiting the slightly acidic microenvironment and the surplus production of H_2_O_2_, resulting in (⋅OH) generation and inducing ferroptosis in tumor cells. To advance the efficiency of Fenton reactions in producing (⋅OH), the same research group co-encapsulated native glucose oxidase (GOD) and ultrasmall Fe_3_O_4_ nanoparticles (NPs) within large, biodegradable dendritic silica NPs with spacious pores 1 year later. GOD facilitated glucose decomposition to generate H_2_O_2_, which actively participated in Fenton reactions with Fe_3_O_4_, thereby significantly enhancing ferroptosis within tumors (Huo et al., 2017). Besides iron, metals such as copper, manganese, and molybdenum are also capable of undergoing Fenton-like reactions to generate (⋅OH), thus initiating ferroptosis (Wu et al., 2019; Wang et al., 2018; Wu C. et al., 2021; He et al., 2023; Li et al., 2022).

#### 4.2.2 GSH depletion and GPx4 inactivation

An alternative approach to induce ferroptosis using NDDSs involves depleting GSH and/or inhibiting GPx4 activity (Figure 12). Researchers from Zhejiang University, led by Wang et al., developed a ferroptosis-inducing agent based on arginine-rich manganese silicate nanobubbles (AMSNs) (Wang et al., 2018). AMSNs have a significant capacity for GSH depletion, thus triggering ferroptosis through GPx4 inactivation. Remarkable antineoplastic effects were observed in both *in vitro* and *in vivo* experiments. At Tianjin University, Meng and collaborators synthesized a metal-organic framework nanocarrier by coordinating a disulfide-bearing imidazole ligand with zinc, encapsulating the photosensitizer (chlorin e6/Ce6) within this carrier. This innovative NDDS induced intracellular GSH depletion via a disulfide-thiol exchange reaction in a murine breast cancer cell line. The depletion of GSH subsequently resulted in GPx4 inactivation and increased cytotoxicity, which could be mitigated by ferroptosis inhibitors (Meng et al., 2019). Low-density lipoprotein docosahexaenoic acid (LDL-DHA) NPs have the capability to induce GSH depletion and GPx4 inactivation in both rat and human liver cancer cells. Concurrently, research indicates that GPx4 plays a central role in LDL-DHA-induced cytotoxicity against tumor cells (Ou et al., 2017).

#### 4.2.3 Direct triggering of ferroptosis

NDDSs can be intentionally engineered to function as carriers for the targeted delivery of molecules capable of inducing ferroptosis (Figure 12), thereby enhancing their potential therapeutic applications. Kim et al. developed ultrasmall silica NPs coated with poly (ethylene glycol), with a diameter measuring less than 10 nm. These NPs, customized with melanoma-targeting peptides, demonstrated the ability to induce ferroptosis in both starved cancer cells and cancer-bearing mice (Kim et al., 2016). Furthermore, a metal-organic network was devised, incorporating an enclosed p53 plasmid, to selectively eliminate cancer cells through a combined ferroptosis/apoptosis mechanism (Zheng et al., 2017). Similarly, Gao et al. designed triggered ferroptotic polymer micelles, composed of an arachidonic acid-conjugated amphiphilic copolymer, which enables rapid cargo release upon free radical triggering in the TME (Gao et al., 2019). This research introduces a novel strategy for overcoming multidrug resistance in cancer through tailored ferroptotic micelles, offering new approaches for managing resistant tumors. Furthermore, Li et al. developed a novel NDDS termed NP ferritin-bound erastin and rapamycin, which exhibited a potent capability to induce ferroptosis by suppressing GPx4 activity and accumulating lipid peroxidation (Li Y. et al., 2019). It is noteworthy that gold NPs loaded with salinomycin have shown the ability to trigger ferroptosis in breast cancer stem cells. This induction occurs through the generation of oxidative stress, mitochondrial dysfunction, and lipid oxidation (Zhao et al., 2019).

#### 4.2.4 Combined therapy

To enhance therapeutic effectiveness, extensive research has been conducted on NDDS-based combination therapies in diverse preclinical models. iu and team presented a novel approach involving biodegradable biomimetic nanospheres composed of copper and manganese silicate. These nanospheres were designed for synergistic chemodynamic/photodynamic therapy, amplifying ferroptosis through the utilization of laser-induced ROS production and the GSH-activated Fenton reaction (Liu et al., 2019). In the pursuit of achieving a synergistic effect between ferroptosis and immunomodulation in cancer treatment, Zhang et al. constructed a biomimetic magnetosome. Comprising a core of Fe_3_O_4_ magnetic nanocluster (NC) and a protective outer layer crafted from pre-engineered leukocyte membranes, this magnetosome is designed for enhanced functionality. Within this framework, the membrane can encapsulate the transforming growth factor-beta inhibitor (Ti) internally, while the PCD protein one antibody (Pa) can be tethered to its outer surface (Zhang F. et al., 2019). Upon intravenous administration, the membrane’s camouflage properties ensure an extended circulation time. Simultaneously, the core of the NC, possessing magnetization and superparamagnetism, facilitates accurate magnetic targeting guided by magnetic resonance imaging. Upon arrival at the tumor site, the combined action of Pa and Ti fosters the development of an immunogenic microenvironment. This microenvironment enhances the H_2_O_2_ levels in polarized M1 macrophages, consequently promoting the Fenton reaction through iron ions released from the NCs. The resultant generation of hydroxyl radicals (⋅OH) triggers fatal ferroptosis in tumor cells. This process also exposes tumor antigens, further augmenting the immunogenicity of the microenvironment. Interestingly, a multifunctional carbon monoxide/thermo/chemotherapy nanoplatform has been reported to increase carbon monoxide-induced ferroptosis. This platform comprises mesoporous carbon NPs as near-infrared-responsive drug carriers, doxorubicin as a chemotherapeutic drug, and triiron dodecacarbonyl as a thermosensitive carbon monoxide prodrug (Yao et al., 2019).

#### 4.2.5 Suppressing ferroptosis

Examination of Figures 11A, B reveals that current research on “ferroptosis-based NDDSs” primarily focuses on treating malignant tumors by inducing ferroptosis in tumor cells, notably in liver cancer, pancreatic carcinoma, and glioma. However, there is a significant scarcity of research on NDDSs engineered to attenuate ferroptosis, as indicated in Figure 12. Herein, we discuss several NDDSs previously documented for their proficiency in inhibiting ferroptosis. The first one involves carboxyl-modified polystyrene NPs, which efficiently hinder ferroptosis by reducing intracellular ROS levels (Li L. et al., 2019). The second approach involves treating cells with deferoxamine (DEF)-hemin and linking them to hydrophilic carbon clusters through polyethylene glycol, a type of NPs that binds to DEF. This strategy affords cellular safeguarding against ferroptosis and senescence (Dharmalingam et al., 2020). The third solution involves the supramolecular self-assembly of an epigallocatechin-3-gallate-selenomethionine nanodrug. This approach effectively alleviates metabolic disorders in chondrocytes and addresses various pathological processes initiated by ferroptosis (Yu H. et al., 2024). In an early 2023 investigation, Liu et al. showcased the capacity of melanin NPs to mitigate sepsis-induced myocardial injury through the suppression of the ferroptosis signaling pathway associated with ROS (Lin et al., 2023b). In May of that year, Yang et al. introduced a novel treatment strategy using polydopamine NPs to address lower back pain resulting from intervertebral disc degeneration. The objective of this approach is to combat ferroptosis by depleting ROS, binding iron ions, and inhibiting GPx4 ubiquitination (Yang X. et al., 2023). In November 2023, a team of researchers at Huazhong Agricultural University disclosed a straightforward one-pot synthesis approach for producing ultrasmall manganese oxide (Mn_3_O_4_) nanoparticles coated with poly (acrylic) acid (Shan et al., 2023). Their findings hold potential for the clinical management of acute liver injuries associated with ferroptosis. Simultaneously, Lan et al. engineered self-assembled NPs loaded with a miR-134-5p inhibitor. These NPs have the ability to target pulmonary epithelial cells and have proven effective in mitigating bronchopulmonary dysplasia by inhibiting ferroptosis, both in *in vivo* and *in vitro* settings (Lan et al., 2023). Additionally, in late 2023, Wang et al. designed and prepared a multifunctional double selenium nanosphere (Wang et al., 2023d). Their work proposes a novel strategy to ameliorate Alzheimer’s disease by inhibiting neural ferroptosis. During the same period, a research team from Dalian Medical University synthesized apigenin-7-glucoside-loaded NPs with the potential to alleviate intestinal ischemia-reperfusion injury by inhibiting ferroptosis (Zhao et al., 2023). In May 2024, Xu et al. designed a macrophage membrane-coated, curcumin-loaded biomimetic nanosponge system aimed at alleviating osteoarthritis by synergistically suppressing inflammation and ferroptosis (Xu et al., 2024). It is important to note that there have been rapid advancements in the field of ferroptosis, continuously uncovering new underlying mechanisms. Consequently, additional research efforts are necessary to expedite the progress of clinical applications employing NDDSs for the specific targeting and inhibition of ferroptosis.

### 4.3 Challenges and future vistas

Despite notable progress in the development of NDDSs capable of inducing or inhibiting ferroptosis, there are ongoing and critical challenges that demand immediate attention moving forward.

#### 4.3.1 Ferroptosis-based NDDSs: A double-edged sword

As discussed earlier, the distinctive ferroptotic attribute corresponds to the ancient Chinese philosophical concept of Yin and Yang (Figure 2B). A matter of concern arises from the dual nature of ferroptosis observed in the context of treating oncological disorders. Intracellular iron serves as the foundation for ferroptosis. With the advancements in nanotechnology, various iron-based nanomaterials, such as liposomes, amorphous iron NPs, biomimetic NPs, and organic iron frameworks, have shown promising therapeutic advantages. This is primarily attributed to their capacity for delivering exogenous iron, which in turn induces tumor ferroptosis (Zhu L. et al., 2022; Jiang et al., 2020; He et al., 2020). However, the excessive use of exogenous metals carries potential risks to human health, including both acute and chronic damage (Zhu L. et al., 2022; Chen Q. et al., 2021; Zheng et al., 2020). Therefore, it is imperative to enhance the precision and dosage control of ferroptosis inducers in cancer therapy, thereby mitigating undesired detrimental effects on healthy tissues (Qiao et al., 2022). Furthermore, there is a need to develop nonferrous NDDSs for the induction of ferroptosis.

#### 4.3.2 The gap between lab research and clinical translation

The significant heterogeneity observed between human diseases and fundamental experimental models represents a major challenge in the field of clinical translation for commercial purposes (Ling et al., 2023; Deng et al., 2022; Cabral et al., 2023). The preparation procedure for most NDDSs centered around ferroptosis is intricate and often involves a variety of biological or chemical constituents. However, contemporary synthesis methodologies face obstacles related to controllable, reproducible, large-scale, cost-efficient, and environmentally sustainable production. These challenges have the potential to lead to detrimental consequences during *in vivo* applications, significantly impeding the commercialization of NDDSs (Zhu S. et al., 2022). NDDSs intended for viable commercial medical applications must exhibit a degree of simplicity, as complex formulations are more likely to experience a high rate of failure during the upscaling of the production process (Darroudi et al., 2021).

While these NDDSs have shown promising results and established safety profiles in cellular and small animal models, a notable gap remains in terms of long-term *in vivo* monitoring, which hinders the translation of these findings into clinical practice (Wang et al., 2023c). Therefore, conducting comprehensive toxicity assessments on NDDSs involving primates and larger animal models becomes essential to ensure a thorough evaluation of safety considerations before clinical implementation (Zhang et al., 2021; Lu et al., 2023; Chen et al., 2023). The dosing regimens applied in human clinical practice deviate from the prevailing strategies observed in animal trials, which usually involve high-dose, short-exposure methods (Su et al., 2018). Furthermore, the presence of physiological, pathological, pharmacological, toxicological, and genetic variations between human subjects and animals adds intricacies to deducing the response of nanomaterials in humans solely based on animal experimentation. Numerous aspects related to the persistence and breakdown products of nanomaterials within the human body remain undiscovered, with a specific focus on genotoxicity, carcinogenicity, and reproductive and developmental toxicity.

Nevertheless, it is crucial to emphasize that only a minute proportion of research endeavors ultimately address the challenges in question, and a substantial number of NDDSs may never advance beyond the preclinical stage or transition to clinical trials (Wang et al., 2023c; Duncan and Gaspar, 2011; Parodi et al., 2022).

#### 4.3.3 Multipronged approaches targeting various novel PCDs

As research advances, emerging variations of PCDs are extensively investigated and linked to diverse human pathological processes. Research demonstrate that ferroptosis, categorized within PCD, is not an isolated occurrence but showcases intricate biological connections with other PCDs (such as autophagy (Liu et al., 2020), cuproptosis (Tong et al., 2022), pyroptosis (Zheng et al., 2022), etc.), crucially contributing to maintaining overall homeostasis in numerous pathological scenarios (Zhao et al., 2022; Lou et al., 2022). However, current studies on “ferroptosis-based NDDSs” largely concentrates on a solitary cell death pathway. In this context, our focus lies on ferroptosis, specifically delving into its associations with emerging PCDs to illuminate the intricate interactions among multiple PCD pathways. Comprehending these interplays will offer valuable insights into future research endeavors and the advancement of innovative NDDSs.

##### 4.3.3.1 Autophagy

Despite its differentiation from other types of PCD, the substantial interaction between autophagy and ferroptosis has garnered growing interest (Ru et al., 2023; Cao et al., 2023b; Kang and Tang, 2017; Zhou et al., 2019; Liu et al., 2020). Oxidative stress and derivatives resulting from lipid peroxidation (e.g., malondialdehyde, ROS, and 4-hydroxynonenal) act as potent triggers for autophagy. Conversely, excessive autophagy promotes ferroptosis (Zhou et al., 2019; Liu et al., 2020). Numerous studies within this research domain have commenced (Li et al., 2022; Feng et al., 2022; Zhang R. et al., 2023).

##### 4.3.3.2 Cuproptosis

Copper-mediated cuproptosis involves the depletion of iron-sulfur cluster proteins, regulating both iron homeostasis and ferroptosis sensitivity (Tsvetkov et al., 2022; Terzi et al., 2021). Ruan et al. developed a microbial nanohybrid using *Escherichia coli* and Cu_2_O NPs (Ruan et al., 2023). This nanohybrid was specifically designed: one function was to induce cellular ferroptosis by inactivating GPx4, while another was to trigger cuproptosis via the aggregation of dihydrolipoamide S-acetyltransferase.

##### 4.3.3.3 Pyroptosis

Although differing in characteristics, both ferroptosis and pyroptosis hold significant research value in tumor therapy (Tong et al., 2022; Gao et al., 2022; Niu et al., 2022). Research has unveiled that CD8^+^ T cells, which are immune cells targeting tumors, possess the capacity to concurrently foster and trigger both varieties of cell death (Tang et al., 2020). To elaborate, CD8^+^ T cells release granzyme A, functioning as a cleaving enzyme for gasdermin B, thereby provoking pyroptosis. For another, interferon-γ secreted by CD8^+^ T cells downregulates solute carrier family seven member 11 (SLC7A11), leading to lipid ROS accumulation and the induction of ferroptosis. Several studies investigating NDDSs co-activating ferroptosis and pyroptosis have been conducted in recent years (Jiang et al., 2023; Xu et al., 2023; Zhang L. et al., 2023).

##### 4.3.3.4 Calcicoptosis

Given that cytosolic calcium elevation significantly regulates ferroptosis (Pedrera et al., 2023; Pedrera et al., 2021), calcicoptosis garners specific attention due to pronounced intracellular Ca^2+^ accumulation. This initiates an irreversible transition in calcium signaling from ‘positive regulation’ to ‘reverse destruction’, ultimately leading to cell death (Bai et al., 2022; Hu et al., 2022). The distinctive regulatory mechanism of intracellular Ca^2+^ has intensified attention on versatile nanomaterials based on calcium (such as calcium phosphate, calcium carbonate, calcium peroxide, and hydroxyapatite) (Zhang M. et al., 2019; Zheng P. et al., 2021; Liu et al., 2022; Tan et al., 2021).

##### 4.3.3.5 Disulfidptosis

In February 2023, Liu et al. introduced disulfidptosis, an emerging type of PCD (Liu et al., 2023c). Their study revealed that tumor cells with elevated SLC7A11 expression accumulate abnormal levels of disulfides, like cysteine, during glucose deprivation, leading to disulfide-induced stress. SLC7A11, a pivotal molecule, directly orchestrates processes such as ferroptosis and is excessively expressed in diverse solid tumors, influencing their growth and progression (Koppula et al., 2021; Chen et al., 2021c).

Additional research is needed to investigate the potential interactions between ferroptosis, autophagy, cuproptosis, pyroptosis, calcicoptosis, and disulfidptosis, with the goal of synergistically targeting multiple forms of PCDs using NDDSs. This comprehensive understanding of the interconnectedness among these distinct PCD mechanisms could be pivotal in overcoming therapeutic resistance.

#### 4.3.4 Artificial intelligence (AI)-Based NDDSs discovery strategies

Currently, the primary obstacle hindering the widespread utilization of NDDSs in pharmaceutical manufacturing is their limited clinical translational capacity (Chan, 2017; Serov and Vinogradov, 2022). Achieving precise and targeted delivery of nanomedicine to affected tissues poses a substantial challenge. This complexity arises from the intricate interplay of multiple variables governing the design, formulation, testing, and selection of NDDSs tailored to specific disease targets. Consequently, it becomes imperative to embrace a fundamentally distinctive approach that emphasizes both the desired attributes and the intended applications in the quest to uncover structure-activity relationships within NDDSs. Such an approach is poised to facilitate the rational design of nanomaterials, providing meticulous control over their physicochemical and biological characteristics (Serov and Vinogradov, 2022).

AI and nanomedicine have recently become closely intertwined (Greenberg et al., 2023). AI provides formidable predictive capabilities, serving as a valuable guide in the rational design of engineered NDDSs tailored for precision drug delivery. Computational modeling and AI offer effective means for analyzing extensive multivariate datasets within limited timeframes. These computational approaches assist in formulating NDDSs, elucidating interactions between NDDSs and biological systems, and constructing models based on high-throughput screening techniques to optimize NDDS selection. The application of nanomaterials in medicine relies heavily on nanotoxicological assessments to ensure their safe use in living organisms. Analyzing and interpreting extensive toxicological data, which includes information from toxicological databases and datasets from high-content image-based screening, is significantly aided by the involvement of AI and machine learning. Physiologically based pharmacokinetic models, alongside nano-quantitative structure-activity relationship models, are utilized to predict the behavior and toxicological effects of nanomaterials, respectively (Singh et al., 2023; Singh et al., 2020).

In the imminent era, the integration of high-throughput and automated systems in the synthesis, characterization, and testing of NDDSs is anticipated to expand, encompassing broader NP libraries. The establishment of comprehensive repositories housing data related to NDDSs libraries, encompassing their relevant physicochemical characteristics, functional evaluations, and *in vivo* delivery statistics, will notably enhance the efficiency of computational models employed to predict the performance of NDDSs. This transformation is poised to usher in an era where the majority of the design and optimization processes for NDDSs are carried out using computational methods. Such a shift in paradigm is expected to significantly abbreviate the time required to identify top-performing NDDSs tailored to specific diseases, thereby streamlining the development of more potent nanotherapeutics (Singh et al., 2023; Hamilton and Kingston, 2023).

Nevertheless, the widespread integration of AI and related algorithms, including machine learning, into nanomedicine faces potential hurdles due to the absence of publicly accessible databases in this field (Wang et al., 2023c). Surmounting challenges, including ensuring data accessibility and sharing, optimizing and validating algorithms, and addressing ethical and privacy considerations, is critical. Despite these impediments, the convergence of AI and NDDSs presents significant developmental opportunities, underscoring the need for additional research and collaborative endeavors to overcome these challenges.

#### 4.3.5 Addressing regulatory, funding, and ethical challenges

“Ferroptosis-based NDDSs” have significant potential to transform clinical practices with more targeted and effective therapies. However, they present challenges that require updated regulatory frameworks, funding strategies, and ethical considerations. 1. Regulatory Frameworks: Developing “Ferroptosis-based NDDSs” requires new regulatory guidelines to address their unique mechanisms. Existing frameworks may not fully address the complexities of ferroptosis and its impact on different cell types. Updated guidelines for clinical trials, safety assessments, and efficacy evaluations specific to ferroptosis-based therapies are needed to ensure thorough testing before patient use. 2. Funding Strategies: “Ferroptosis-based NDDSs” are likely to attract interest from both public and private sectors. Effective funding mechanisms and incentives are essential for advancing research and development. Sufficient funding can accelerate the translation of discoveries into clinical applications, enhance innovation, and support the development and market introduction of promising therapies. 3. Ethical Considerations: As ferroptosis-based therapies advance, it is crucial to address ethical issues such as equitable access and affordability. Policies ensuring that new treatments are accessible to diverse populations and fairly priced are vital for preventing healthcare disparities and ensuring that advancements benefit all patients.

#### 4.3.6 Others

Our current comprehension of ferroptosis in human diseases likely represents only a fraction of the overall scope, and research in this domain remains in its nascent stages. Importantly, the sensitivity to ferroptosis differs across different cell types and developmental stages. This sensitivity is chiefly determined by the underlying metabolic state of cells, including elements such as iron concentrations, amino acid accessibility, lipid metabolism, mitochondrial performance, and other complex metabolic pathways. Even minor modifications in crucial molecules can exert a substantial influence on cellular vulnerability to ferroptosis. Consequently, it becomes imperative to account for individual variations when devising strategies for the design of NDDSs (Wang et al., 2022d).

### 4.4 Strengths and limitations

Contrary to previous inquiries, predominantly confined to systematic or narrative reviews, combining scientometrics and visualized analysis offers readers a clearer depiction of research focal points and trends across various dimensions (Zhang et al., 2022b). Representing the initial bibliometric analysis that seeks to chart the knowledge terrain of “ferroptosis-based NDDSs” over the past 11 years, this investigation offers a relatively comprehensive and impartial reference, despite inherent limitations.

This study has several limitations: 1) Utilizing CiteSpace, limited to WoSCC publications, may introduce selection bias due to software limitations (Ling et al., 2023). 2) Depending on citation counts, influenced by variables like publication date and journal quality, may not precisely reflect a paper’s impact. 3) Inability to comprehensively review all papers and subdomains necessitated equal attention to both high and low-quality publications, potentially affecting reliability. 4) Scientometric methodologies, rooted in natural language processing, may exhibit biases, as observed in prior studies (Cao et al., 2023a; Zhang J. et al., 2022; Peng et al., 2022; Cao et al., 2024a; Cao et al., 2024b; Cao et al., 2024c; Cao et al., 2024d; Cao et al., 2024e). 5) Limiting the study to English documents might introduce publication bias. 6) Incomplete retrieval of recent literature and keywords could impact results due to gaps in literature collection. 7) VOSviewer automatically extracts author names, which may not always be accurate. Some authors may use different spellings or multiple names, potentially leading to inaccuracies in research results for these authors (Peng et al., 2022).

## 5 Conclusion

There is currently a significant gap in scientometric analyses of “ferroptosis-based NDDSs”. This pioneering study offers the first comprehensive scientific metrology study of “ferroptosis-based NDDSs” in the biomedical domain over the past 11 years. The main findings are: 1) Bibliometric assessments have identified significant advancements in “ferroptosis-based NDDSs”, highlighting key contributing nations, institutions, researchers, and central research themes. 2) Co-citation and keyword analyses have highlighted influential studies, emerging patterns, and key research areas in the ferroptosis domain, offering a roadmap for future research directions. 3) The significant challenges in the clinical translation of “ferroptosis-based NDDSs” have been discussed, emphasizing the need for interdisciplinary collaboration and the urgency of overcoming technical and regulatory obstacles. Addressing these challenges is essential for realizing the full potential of NDDSs in revolutionizing healthcare. These breakthroughs are crucial for enhancing the scientific community’s ability to accurately identify and address existing challenges while fostering new ideas. These findings enable the research community to identify emerging themes and frontiers that will guide and propel the advancement of “ferroptosis-based NDDSs”. In conclusion, the focus on ferroptosis marks a groundbreaking chapter for NDDSs, promising to transform healthcare despite existing challenges. Increased efforts and stronger collaboration across chemistry, materials science, toxicology, biology, basic science, and clinical medicine are vital to overcoming these obstacles and unleashing the full potential of “ferroptosis-based NDDSs” in the biomedical domain.

## Data Availability

The raw data supporting the conclusions of this article will be made available by the authors, without undue reservation.
