# Supplementary material for: Mapping the evolution and research landscape of ferroptosis-targeted nanomedicine: insights from a scientometric analysis
Source: Front Pharmacol. 2024 Sep 25;15:1477938. doi: 10.3389/fphar.2024.1477938 (PMC11461269; doi:10.3389/fphar.2024.1477938)
Supplement: Supplementary file 1 [file Table1.DOCX]

Supplementary Material

# Search strategy

| **Search** | **Query** |
| --- | --- |
| #1 | TS=(“ferroptosis” OR “iron death” OR “iron overload”) |
| #2 | TS=("Nanoparticle Drug Delivery System" OR "Nanoparticle Based Drug Delivery System" OR "Nano Delivery System" OR "Delivery System, Nano" OR "Delivery Systems, Nano" OR "Nano Delivery Systems" OR "System, Nano Delivery" OR "Systems, Nano Delivery" OR "Nano Drug Delivery Systems" OR "Nano-Drug Delivery System" OR "Delivery System, Nano-Drug" OR "Delivery Systems, Nano-Drug" OR "Nano Drug Delivery System" OR "System, Nano-Drug Delivery" OR "Systems, Nano-Drug Delivery" OR "nanoparticle" OR "nanoparticles" OR "biomimetic nanoparticle" OR "biomimetic nanoparticles" OR "metallic nanoparticle" OR "metallic nanoparticles" OR "inorganic nanoparticle" OR "inorganic nanoparticles" OR "Nanocrystalline Materials" OR "Material Nanocrystalline" OR "Materials Nanocrystalline" OR "Nanocrystalline Material" OR "Nanocrystals" OR "Nanocrystal" OR "Micelles" OR "Polymer Micelles" OR "Polymer Micelle" OR "Micel" OR "micella" OR "Transferosomes" OR "Transferosome" OR "Metal-Organic Frameworks" OR "Frameworks, Metal-Organic" OR "Metal Organic Frameworks" OR "Porous Coordination Networks" OR "Coordination Networks, Porous" OR "Networks, Porous Coordination" OR "Porous Coordination Polymers" OR "Coordination Polymers, Porous" OR "Polymers, Porous Coordination" OR "Porous Coordination Polymer" OR "Coordination Polymer, Porous" OR "Polymer, Porous Coordination" OR "Metal Organic Framework" OR "Framework, Metal Organic" OR "Organic Framework, Metal" OR "Metal-Organic Framework" OR "Framework, Metal-Organic" OR "Covalent Organic Framework" OR "Framework, Covalent Organic" OR "Organic Framework, Covalent" OR "Nanoflower" OR "Nanoflowers" OR "Nanozymes" OR "nanozyme" OR "Liposomes" OR "Liposome" OR "Transferosomes" OR "Transferosome" OR "Ultradeformable Liposomes" OR "Liposome, Ultradeformable" OR "Liposomes, Ultradeformable" OR "Ultradeformable Liposome" OR "Liposomes, Ultra-deformable" OR "Liposome, Ultra-deformable" OR "Liposomes, Ultra deformable" OR "Ultra-deformable Liposome" OR "Ultra-deformable Liposomes" OR "Niosomes" OR "Niosome" OR "metal organic clusters" OR "nanocage" OR "Mesoporous Silica" OR "nano tubes" OR "nano tube" OR "Nanomedicine" OR "Nanogels" OR "Nanogel" OR "Nanocomposite Gels" OR "Nanocomposite Gel" OR "Gel, Nanocomposite" OR "Nanocomposite Hydrogels" OR "Nanocomposite Hydrogel" OR "Hydrogel, Nanocomposite" OR "Dendrimer") |
| #3 | #1 AND #2 |
